# Transglutaminase 2 promotes tumorigenicity of colon cancer cells by inactivation of the tumor suppressor p53

**DOI:** 10.1038/s41388-021-01847-w

**Published:** 2021-06-08

**Authors:** Patrizia Malkomes, Ilaria Lunger, Elsie Oppermann, Khalil Abou-El-Ardat, Thomas Oellerich, Stefan Günther, Can Canbulat, Sabrina Bothur, Frank Schnütgen, Weijia Yu, Susanne Wingert, Nadine Haetscher, Claudia Catapano, Marina S. Dietz, Mike Heilemann, Hans-Michael Kvasnicka, Katharina Holzer, Hubert Serve, Wolf Otto Bechstein, Michael A. Rieger

**Affiliations:** 1 Goethe University Hospital Frankfurt, Department of General, Visceral and Transplant Surgery, Frankfurt am Main, Germany; 2 Goethe University Hospital Frankfurt, Department of Medicine, Hematology/Oncology, Frankfurt am Main, Germany; 3 German Cancer Consortium and German Cancer Research Center (DKFZ), Heidelberg, Germany; 4 Frankfurt Cancer Institute, Frankfurt am Main, Germany; 5 Max Planck Institute for Heart and Lung Research, Department I Cardiac Development and Remodelling, Bad Nauheim, Germany; 6 Single Molecule Biophysics, Institute of Physical and Theoretical Chemistry, Goethe University Frankfurt, Frankfurt am Main, Germany; 7 Goethe University Frankfurt, Senckenberg Institute for Pathology, Frankfurt am Main, Germany; 8 Cardio-Pulmonary Institute, Frankfurt am Main, Germany; 9 Present Address: Philipps University of Marburg, Department of Visceral-, Thoracic- and Vascular Surgery, Marburg, Germany

**Keywords:** Colorectal cancer, Oncogenes

## Abstract

Despite a high clinical need for the treatment of colorectal carcinoma (CRC) as the second leading cause of cancer-related deaths, targeted therapies are still limited. The multifunctional enzyme Transglutaminase 2 (TGM2), which harbors transamidation and GTPase activity, has been implicated in the development and progression of different types of human cancers. However, the mechanism and role of TGM2 in colorectal cancer are poorly understood. Here, we present TGM2 as a promising drug target.

In primary patient material of CRC patients, we detected an increased expression and enzymatic activity of TGM2 in colon cancer tissue in comparison to matched normal colon mucosa cells. The genetic ablation of TGM2 in CRC cell lines using shRNAs or CRISPR/Cas9 inhibited cell expansion and tumorsphere formation. In vivo, tumor initiation and growth were reduced upon genetic knockdown of TGM2 in xenotransplantations. TGM2 ablation led to the induction of Caspase-3-driven apoptosis in CRC cells. Functional rescue experiments with TGM2 variants revealed that the transamidation activity is critical for the pro-survival function of TGM2. Transcriptomic and protein–protein interaction analyses applying various methods including super-resolution and time-lapse microscopy showed that TGM2 directly binds to the tumor suppressor p53, leading to its inactivation and escape of apoptosis induction.

We demonstrate here that TGM2 is an essential survival factor in CRC, highlighting the therapeutic potential of TGM2 inhibitors in CRC patients with high TGM2 expression. The inactivation of p53 by TGM2 binding indicates a general anti-apoptotic function, which may be relevant in cancers beyond CRC.

## Introduction

Colorectal cancer (CRC) is the third leading cancer worldwide, and the second leading cause of cancer-related deaths [1]. At least 20% of patients present initially with metastatic disease, and 25–30% of patients with UICC stage II/III disease will ultimately develop recurrence within 5 years after surgery [2]. During the past decade, improved surgical techniques and new adjuvant therapy regimens significantly increased the survival. However, the 5-year survival rate of all tumor stages is still less than 60% and CRC patients with advanced disease continue to have a poor prognosis [3–5]. Despite a comprehensive understanding of the molecular sequence of events in colorectal carcinogenesis [6, 7], effective targeted therapy options are lagging behind compared to other cancers [5, 8]. Globally, more than half a million CRC patients succumb every year. There is a tremendous need for improved therapies in CRC, and new targets need to be defined in order to develop specific and effective drugs for long-term CRC management.

Transglutaminase 2 (TGM2) belongs to the family of tissue transglutaminases. All enzymatic active transglutaminases have a catalytic triad at the active site in common, consisting of a cysteine, histidine, and aspartate residue, and catalyze post-translational modifications of proteins in a Calcium-dependent manner [9–13], thereby generating protein–protein crosslinks. Although all mammalian forms of transglutaminases have perceptible structural homology and several features in common, they differ in their tissue distribution and localization as well as in the mechanism of action and substrate specificity [14–16].

Besides the well-characterized transamidase (TGase) activity resulting in protein cross-linking, TGM2 can function as a G-protein coupled membrane receptor (GTPase) [17]. Both enzymatic activities are mutually exclusive and are mainly regulated by conformational changes upon Calcium or GTP-binding to the C-terminal domain of TGM2 [18, 19]. Due to its diverse enzymatic activities, TGM2 has been implicated in many biological processes such as inflammation [20], wound healing [21], cell survival, and apoptosis [22], as well as in the pathophysiology of various diseases such as celiac disease [23], neurodegenerative disorders [24], and cancer [25]. In various cancer entities, an implication of TGM2 in cancer development, progression, metastasis, and cancer stem cell maintenance has long been discussed [26, 27]. Although other groups have studied the role of TGM2 in colon cancer, the results are controversial, partly due to the pleiotropic functions of TGM2. Fernández-Acenero et al. found that high TGM2 expression in the stroma is associated with increased relapse risk in CRC, showing the potential of TGM2 as a prognostic marker [28]. It remains uncertain, whether TGM2 has a pro- or antitumorigenic role [29, 30], if it promotes tumor cell survival or apoptosis [31, 32], and whether epithelial–mesenchymal transition and invasion are supported by TGM2 [33–36]. To resolve some of these long-standing questions, we determined the expression and activity of TGM2 in primary patient material and accurately investigated the functional mechanism of TGM2 in CRC including preclinical mouse models and mechanistic in vitro studies at single cell level. We observed that TGM2 expression was upregulated in CRC tissue in comparison to adjacent normal colon tissue. TGM2 enhanced the tumorigenicity of colon cancer cells by inactivation of the tumor suppressor p53 and thus mediates escape of apoptosis induction. Our data demonstrate that TGM2 is essential for CRC cell survival by direct interaction with p53 that might be involved in the pathogenesis of colon cancer.

## Results

### TGM2 protein expression and enzymatic activity are elevated in CRC

**Fig. 1:**
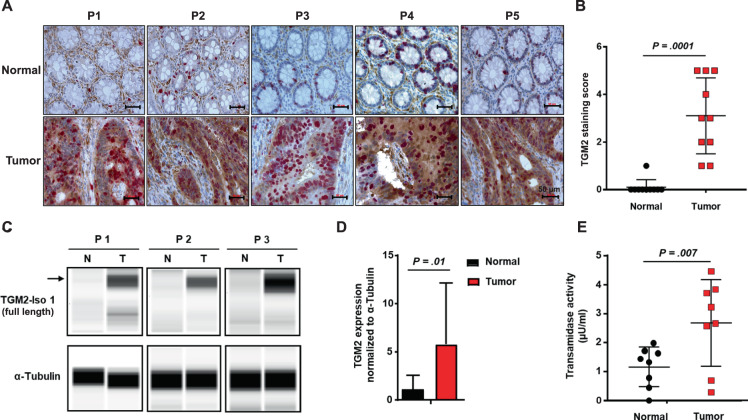
TGM2 protein expression and enzymatic activity are elevated in CRC. **A** Representative microphotographs of immunohistochemical (IHC) stainings of TGM2 (brown) and Ki67 (red) in paired tumor and corresponding normal tissues from five CRC patients (P1-5). Scale bar, 50 µm. **B** Comparison of TGM2 expression in 10 colon cancer and paired adjacent noncancerous colon tissues. Data are presented as mean immunoreactive score ± SD of IHC staining. **C** Representative protein expression data of TGM2-isoform 1 in primary patient material showing normal tissue (N) versus the corresponding tumor tissue (T) of three different CRC patients (P1-3) via Simple Western technology. α-Tubulin served as loading control. **D** Quantification of TGM2 protein expression in epithelial cells of paired tumor/normal tissue samples from CRC patients (*n* = 8) via Simple Western technology. **E** TGM2 transamidation activity in epithelial cells of paired tumor/normal tissue samples from CRC patients (*n* = 8). Results are presented as mean ± SD. Pairwise comparisons were performed using Wilcoxon matched pairs test.

We first assessed TGM2 protein expression in CRC and matched normal colon mucosa tissues via immune staining in histological specimens of ten CRC patients. After confirming the specificity of the monoclonal antibody against TGM2 (Supplementary Fig. S1), we demonstrated that TGM2 was expressed to a higher extent in epithelial cancer tissue than in matched normal colon epithelium in all investigated cases (Fig. 1A, B, Supplementary Fig. S2). Patient and tumor characteristics of all selected cases are shown in Supplementary Table S1. In addition to cytoplasmic TGM2 staining, we observed nuclear localization of TGM2 in tumor cells (Supplementary Fig. S3).

To further quantify the TGM2 protein expression level in tumor versus corresponding normal colon mucosa, freshly resected tissue specimens from CRC patients undergoing surgical resection of their primary tumor were investigated. After enrichment of epithelial cells from these pathological classified tissues, we quantitatively assessed TGM2 protein expression via Simple Western technology (Fig. 1C). In eight investigated pairs, TGM2 was always expressed to a higher extent in the tumor (at least 2-fold, up to 16-fold, *P* = 0.01) in comparison to the corresponding normal colon epithelium (Fig. 1D). Next, we determined the transamidase activity (TGase) in tumor/normal epithelium. Again, we found a significantly enhanced TGase activity in the tumor cells (Fig. 1E).

### TGM2 is essential for cancer cell expansion and tumor initiation

**Fig. 2:**
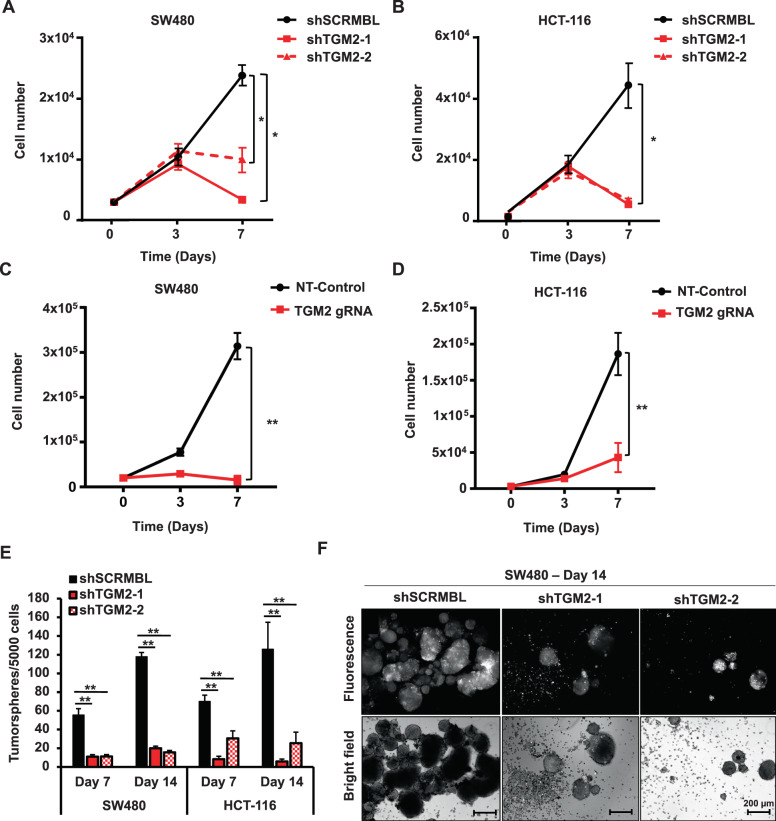
Targeting TGM2 inhibits CRC cell expansion and tumorsphere formation. **A** Cell expansion of lentivirally transduced SW480 or **(B)** HCT-116 cells with two independent shRNAs against TGM2 (shTGM2-1, shTGM2-2) or control (shSCRMBL). **C** Cell expansion of SW480 and **(D)** HCT-116 cells after transduction with CRISPR/Cas9 constructs (TGM2 gRNA) against TGM2 or non-target (NT) control. **E** Mean number of tumorspheres after TGM2 knockdown in SW480 and HCT-116 cells. Data are presented as mean ± SD of at least three independent experiments. **F** Representative microphotographs of tumorspheres of SW480 cells 14 days after transduction with shTGM2-1, shTGM2-2, or control (shSCRMBL). Shown are fluorescent (tdTOMATO) and brightfield microphotographs. Scale bar, 200 µm. *P < 0.05; **P < 0.01, Mann–Whitney *U* test.

The accelerated TGM2 expression and activity in cancer tissue prompted us to investigate the functional significance of TGM2 upregulation in CRC. We genetically knocked down TGM2 in CRC cell lines SW480 and HCT-116, both expressing TGM2 at high levels, using two different shRNAs (referred to as shTGM2-1 and shTGM2-2). Both shRNAs showed an efficient TGM2 knockdown (Supplementary Fig. S4A, S4B). The lentiviral transduction was at least 80% of cells in all experiments (Supplementary Fig. S4D). Compared to the scrambled control shRNA (shSCRMBL), both shRNAs against TGM2 severely impaired cell expansion of CRC cells over time (Fig. 2A, B). These results were confirmed using lentiviral CRISPR/Cas9 vectors to genetically knockout TGM2 expression (Supplementary Fig. S4C). Once more, after TGM2 knockout, SW480 and HCT-116 cells were unable to expand in culture (Fig. 2C, D). We next assessed the tumorsphere formation capacity as a surrogate indicator for stem cell activity in both cell lines. TGM2 knockdown almost abolished tumorsphere formation (Fig. 2E, F).

**Fig. 3:**
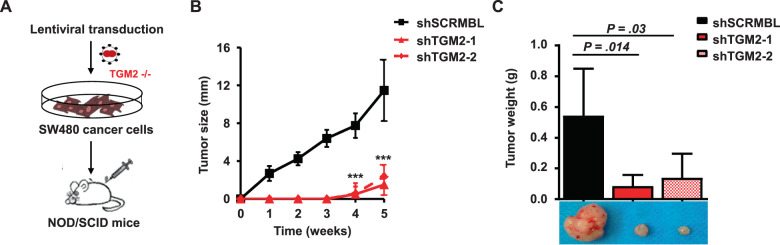
Targeting TGM2 blocks CRC cell tumorigenicity. **A** SW480 cells were transduced with TGM2 shRNAs or control and expanded in vitro for three days. A total of, 50,000 living cells were injected subcutaneously into the flanks of NOD/SCID mice. Tumor volumes were monitored over time and determined using caliper measurements. **B** Comparison of growth curves of xenografts of SW480 transduced with shTGM2-1, shTGM2-2, or shSCRMBL. **C** After five weeks, mice were sacrificed, tumor xenografts were harvested, and tumor weight was measured. Representative tumors are shown. Data are represented as mean ± SD of xenografts in eight mice per group. ***P < 0.001, Mann–Whitney *U* test.

To determine the effect of TGM2 depletion on tumor initiation and progression in vivo, we subcutaneously transplanted SW480 cells with or without TGM2 stably knocked-down into NOD/SCID mice (Fig. 3A). Deficiency in TGM2 resulted in a significant slowdown in tumor initiation and growth over time in vivo (Fig. 3B). After tumor resection, shTGM2-transduced tumors were very small (0.1 g) in comparison to scrambled shRNA-transduced tumors (0.55 g) (Fig. 3C). These results demonstrate an essential function of TGM2 in CRC cell growth and suggest TGM2 as a promising target to inhibit CRC tumor progression.

### TGM2 knockdown induces cell death in CRC via Caspase-3 activation

**Fig. 4:**
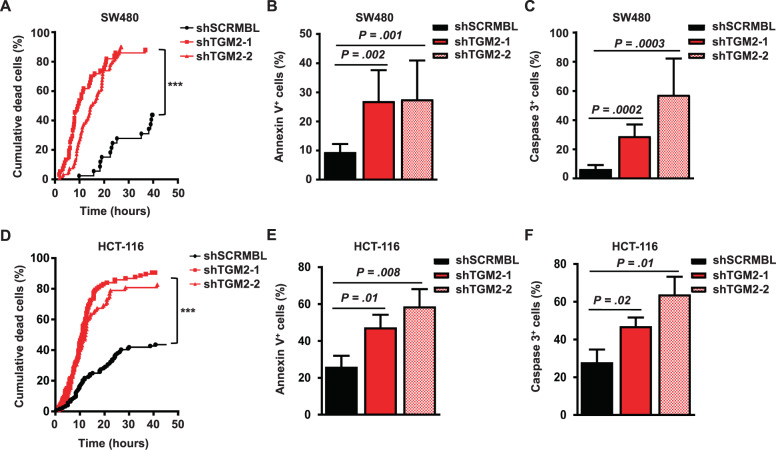
The ablation of TGM2 leads to CRC cell death. **A** Time-lapse imaging of SW480 cells transduced with shTGM2-1, shTGM2-2 or shSCRMBL. Shown are cumulative cell death events over time determined by single cell tracking. *P* value was calculated by log-rank test. **B** Percentage of apoptotic SW480 cells determined by Annexin V/7-AAD staining 72 hours after TGM2 knockdown. **C** Percentage of Caspase-3 positive SW480 cells 72 hours after TGM2 knockdown. **D**–**F** All experiments were repeated in HCT-116 cells transduced with shTGM2-1, shTGM2-2 or shSCRMBL. **D** Time lapse imaging showing the cumulative cell death events. **E** Percentage of Annexin V positive and (**F)** Caspase-3 positive HCT-116 cells 72 hours after TGM2 knockdown. Results are presented as mean ± SD of three independent experiments. ***P < 0.001, Mann–Whitney *U* test.

To assess the fate of CRC cells upon TGM2 inactivation, we investigated the cell behavior induced by TGM2 knockdown continuously at single cell resolution using time-lapse microscopy-based cell tracking [37, 38]. This technology allowed us to follow individual tumor cells and their progeny over many days in culture after lentiviral shRNA transduction. Results have shown that more than 80% of individual SW480 cells died after TGM2 knockdown within 40 hours of observation, without going into cell division, whereas the majority of shSCRMBL-transduced cells divided in the 40-hours time-frame (Fig. 4A). To confirm these results, we performed Annexin V/7-AAD and Annexin V/active Caspase-3 stainings via flow cytometry. 72 hours after lentiviral transduction of TGM2 shRNAs, the percentage of apoptotic cells was massively increased (Fig. 4B, Supplementary Fig. S5A). 30% (shTGM2-1) and 50% (shTGM2-2) of the transduced SW480 cells showed active Caspase-3 positive staining after TGM2 knockdown, in contrast with only 7% active Caspase-3 positive cells in the control (Fig. 4C, Supplementary Fig. S5B). We recapitulated these results using a second CRC cell line HCT-116. As shown in SW480 cells, a knockdown of TGM2 resulted in cell death of more than 80% of transduced HCT-116 cells as detected by single cell tracking (Fig. 4D). Again, we could observe that 72 hours after lentiviral transduction of TGM2 shRNAs, the percentage of apoptotic (Fig. 4E) and Caspase-3 positive cells (Fig. 4F) significantly increased in comparison to shSCRMBL-transduced cells, indicating that the inactivation of TGM2 rapidly leads to the induction of Caspase-3-dependent apoptosis.

### TGM2 transamidase activity is critical for tumor cell growth

**Fig. 5:**
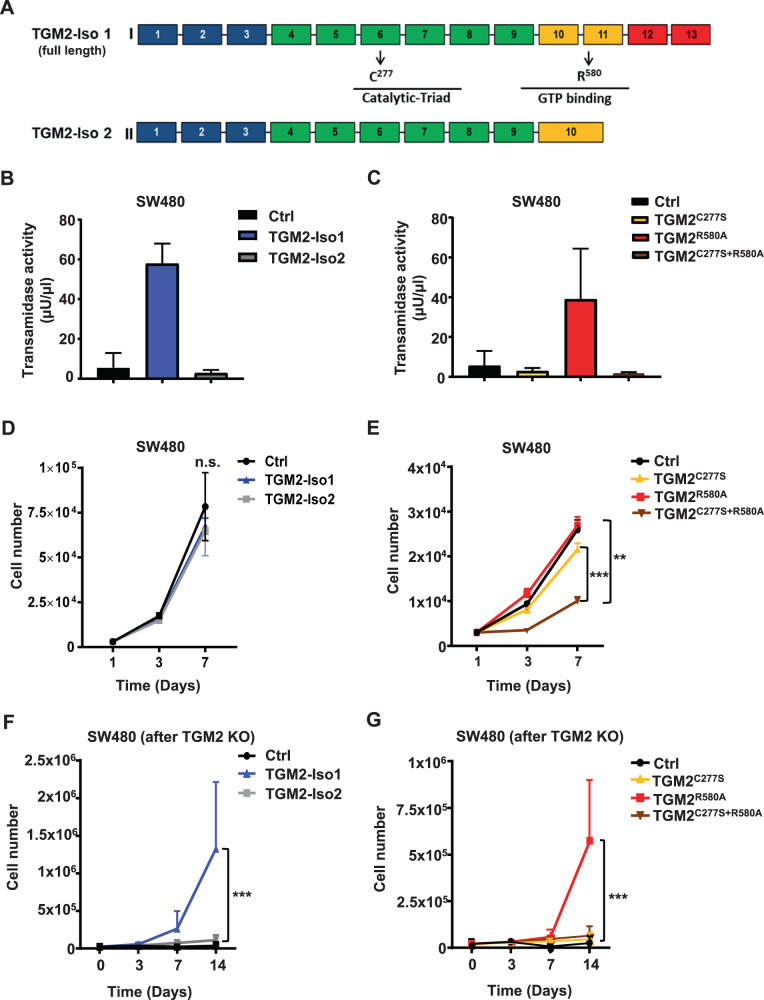
Transamidation activity of TGM2 is predominant in CRC and essential for cancer cell survival. **A** Schematic diagram of full-length TGM2 isoform 1 (I) and short or truncated isoform 2 (II) with respective exons. Positions of point mutants are indicated. **B** Transamidase activity assay with lysates of SW480 cells after lentiviral overexpression of TGM2-isoform 1 or 2, three days after transduction. Data are presented as mean ± SD of three independent experiments. **C** Transamidase activity assay with cell lysates after lentiviral overexpression of TGM2^C277S^, TGM2^R580A^, TGM^C277S+R580A^, or vector control three days after transduction. Data are presented as mean ± SD of three independent experiments. **D** Cell expansion of SW480 cells lentivirally expressing TGM2 isoform 1, 2 (TGM2-Iso 1, TGM2-Iso 2) or vector control (Ctrl) or **(E)** TGM2 mutants TGM2^C277S^, TGM2^R580A^, TGM^C277S+R580A^, or vector control (Ctrl). Cells were counted at day three and seven after transduction. **F** SW480 cells were transduced with TGM2 isoforms 1 or 2, or **(G)** with mutated TGM2 constructs C277S, R580A, or C277S/R580A and expanded for three days. Subsequently, cells were double transduced with CRISPR/Cas TGM2 knockout construct (TGM2 KO) or control, resulting in genetically manipulated cells with only ectopic TGM2. Transduction efficiency was evaluated by FACS and expansion kinetic monitored over 14 days. Results are presented as mean ± SD of three independent experiments. Mann–Whitney *U* test; **P < 0.01; ***P < 0.001.

TGM2 is a bifunctional enzyme with mutually exclusive TGase or GTPase activity. Since the transamidase activity is enhanced in tumor cells versus normal epithelium in CRC, we sought to assess the responsible enzymatic function for the enhanced tumor cell survival. We cloned the open reading frames of TGM2 splice variants 1 (full-length TGM2) and 2 (short form lacking exons 11-13) and point mutated enzymes in a lentiviral expression vector (Fig. 5A). We confirmed the overexpression by Simple Western analysis (Supplementary Fig. S6A) and the TGase activity (Fig. 5B). As previously described, isoform 2 lacks transamidase function [31]. We further generated well-described point mutants of TGM2, C277S (lack of TGase function), R580A (lack of GTPase function), and the enzymatically null double mutant C277S/R580A. The protein expression level of TGM2 was similar with all constructs (Supplementary Fig. S6B), but only the GTPase mutant R580A retains full TGase activity (Fig. 5C). Gain-of-function studies with TGM2 isoforms 1 and 2 in SW480 cells and HCT-116 cells did not alter cell expansion (Fig. 5D, Supplementary Fig. S6C). The overexpression of the mutants C277S and R580A showed no effect on cell expansion, whereas the double mutant C277S/R580A showed a dominant-negative effect in SW480 and HCT-116 cells (Fig. 5E, Supplementary Fig. S6D). To determine which isoform and enzymatic activity is essential for CRC cell expansion, we overexpressed TGM2 isoform 1 and 2 by lentiviral transduction in SW480 cells, followed by a knockout of endogenous TGM2 using CRISPR/Cas9. Only TGM2 full-length isoform 1 was able to rescue the growth disability after TGM2 knockout (Fig. 5F), whereas the shorter isoform 2 did not show any benefit in comparison to the control. Similarly, only the rescue expression by the TGM2 mutant R580A was able to fully restore SW480 cell expansion, neither of the TGase null mutants were able to rescue the cell growth (Fig. 5G). These results highlight the necessity of the TGase functional ability of TGM2 in providing tumor cell survival and expansion in CRC.

To determine whether increased TGM2 levels protect cells from Caspase-3 mediated apoptosis, we overexpressed TGM2 isoform 1 or 2 as well as enzymatic mutants in SW480 cells. The cells were subsequently treated with the chemotoxic agent Oxaliplatin, a strong inducer of Caspase-3. As shown in Supplementary Fig. S7A and S7B, overexpression of the TGase active full-length isoform 1 and the TGase active mutant R580A decreased Caspase-3 activation upon Oxaliplatin treatment in comparison to the control cells or to cells expressing the TGM2 isoform 2 and TGM2 mutants without TGase activity.

### Molecular changes in CRC upon TGM2 knockdown reveals upregulation of p53 signaling

**Fig. 6:**
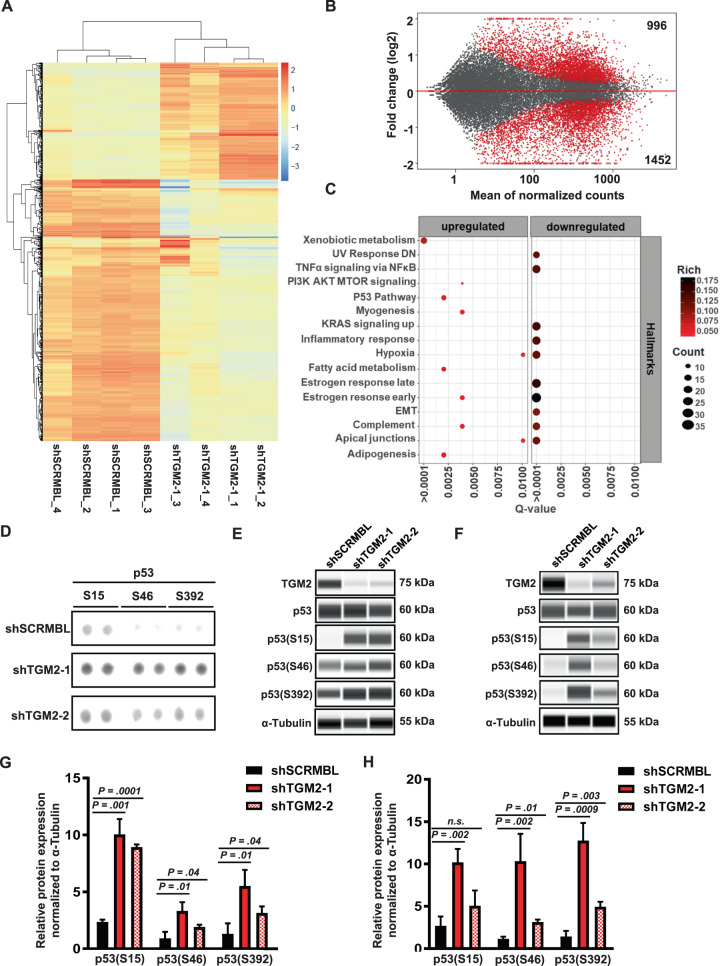
Molecular changes after TGM2 knockdown reveal p53 as central tumor suppressor. **A**–**C** Gene expression profiling by RNA-seq of SW480 cells after transduction with either shTGM2-1 or shSCRMBL. **A** Unsupervised hierarchical clustering of the top 1000 differentially expressed genes (DEGs) upon TGM2 knockdown across the four biological replicates. **B** MA plot relating *p* values for all differentially expressed genes between shTGM2-1 and shSCRMBL from four biological replicates. Red dots indicate significantly regulated genes (adjusted *P* < 0.05). List of regulated genes is presented in Supplementary Table S2. **C** Scatter plot of gene set enrichment analysis of DEGs relating the Q-value for Hallmark gene-set signatures. The top 16 enriched pathways are shown (*P* < 0.05, Fold change ≥2). The color and size of each dot represent the Rich factor and the number of DEGs mapped to the indicated pathway, respectively. **D** Proteome analysis of regulated proteins involved in apoptosis upon shRNA-mediated TGM2 knockdown. Representative blot of Proteome Profiler Array™-Human Apoptosis Array analysis of SW480 cells. The regulation of protein expression of phosphorylated p53 variants is shown. **E**–**H** Quantification of p53 and phosphorylated p53 (S15, S46, and S392) upon TGM2 knockdown in SW480 (**E**, **G**) and HCT-116 (**F**, **H**) cells via Simple Western technology (*n* = 3; Mann–Whitney *U* test).

We further assessed the transcriptional program after TGM2 knockdown in SW480 cells by RNA-seq 48 hours after transduction. RNA-seq revealed profound changes in gene expression upon TGM2 knockdown, with 996 genes being significantly upregulated, and 1452 genes being downregulated (≥2-fold change, adjusted *P* < 0.05) in comparison to shSCRMBL-transduced cells (Fig. 6A, B, Supplementary Table S2). Pathway analyses and gene set enrichment analyses demonstrated alterations in many cancer-related hallmark pathways, such as the downregulation of NFkB signaling, KRAS signaling, inflammatory responses, hypoxia, estrogen response and UV response, and upregulation of p53 signaling, among others (Fig. 6C). RNA-seq of HCT-116 CRC cells upon TGM2 knockdown recapitulated these results, revealing the p53 pathway as the primary regulated pathway upon TGM2 knockdown in various gene set enrichment databases (KEGG and Panther, Supplementary Fig. S8, Supplementary Table S2). Since we showed the rapid induction of Caspase-3-dependent apoptosis upon TGM2 inactivation, we further focused on the p53 pathway. A human protein array for apoptosis pathways confirmed a strong hyperactivation of phosphorylated p53 at various phosphorylation sites including S15, S46, and S392 (Fig. 6D) in SW480 lysates after TGM2 knockdown. These results were further consolidated by protein expression analyses via Simple Western technology in SW480 (Figs. 6E, G) and HCT-116 cells (Figs. 6F, H). Densitometric analysis of the proteome array showed further an increased expression of Procaspase-3 and heat-shock proteins (HSP 32, 27, and 70) in shTGM2-transduced cells in comparison to control-transduced cells (Supplementary Fig. S9A, S9B). The molecular changes induced by TGM2 knockdown, led us to hypothesize that TGM2 specifically regulates essential pathways for tumor cell survival and directly influences a p53-mediated tumor growth suppression in CRC.

### P53-mediated tumor suppression is inactivated by the physical interaction with TGM2

**Fig. 7:**
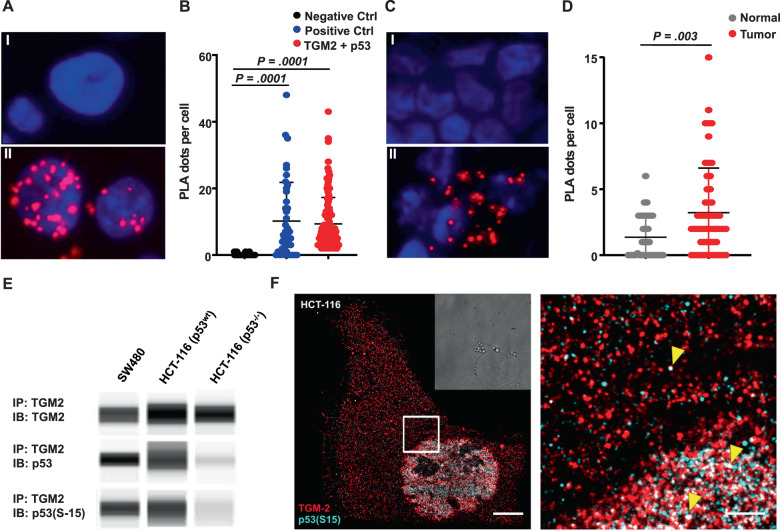
Direct interaction of TGM2 and p53 in CRC. **A** Representative images of proximity ligation assay (PLA) of TGM2 and p53 in SW480 cells. Cells incubated only with TGM2 antibody served as negative control (I). Protein–protein interaction of TGM2 and p53(S15) was visualized using hybridization probes labeled with Texas Red (II). Nuclei were stained with DAPI (blue). **B** Quantification of TGM2-p53 interaction and associated technical controls (Ctrl). Technical controls demonstrate the specificity of PLA signals. Each dot represents one cell. Mean value of PLA dots per cell is shown by the black line. **C** Representative images of proximity ligation assay of TGM2 and p53 in patient-derived normal epithelial cells (I) and corresponding colon cancer cells (II). **D** Quantification of TGM2-p53 interaction in primary patient material. (Significance was calculated using Kruskal–Wallis test). **E** Co-immunoprecipitation (Co-IP) of endogenous TGM2 and p53 or phosphorylated p53(S15) in SW480, HCT-116 p53 wildtype cells (wt) and HCT-116 p53 knockout cells (−/−). **F** Super-resolved image of a HCT-116 cell immunostained for TGM2 (red) and p53(S15) (cyan). A zoom-in of the highlighted region is shown on the right. White regions indicate overlapping signal of TGM2 and p53(S15) (yellow arrowheads). Scale bars represent 5 µm and 1 µm, respectively.

**Fig. 8:**
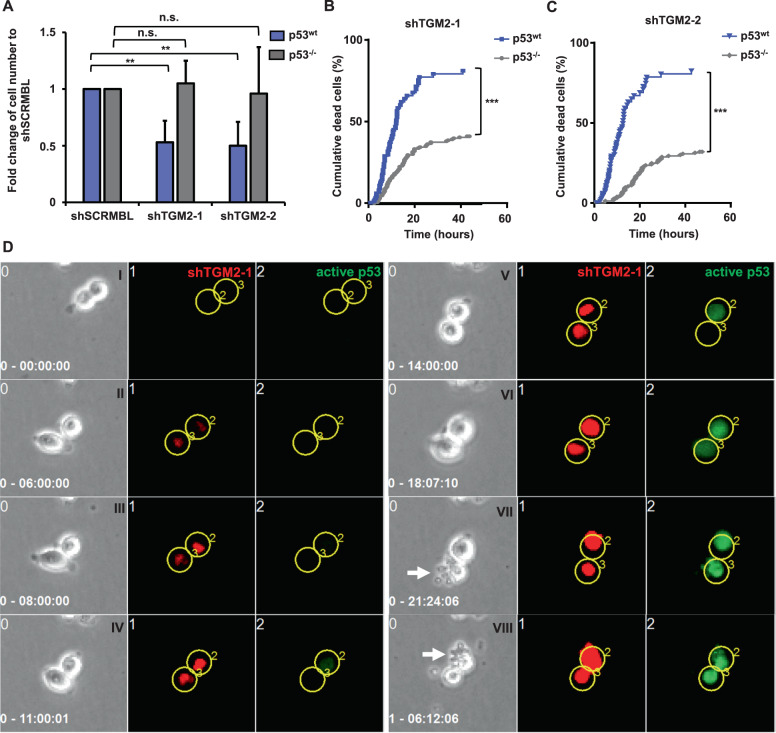
TGM2 functions as a pro-survival molecule via direct interaction and inhibition of p53 signaling. **A**–**C** HCT-116 p53 wildtype cells (wt) and HCT-116 p53 knockout cells (−/−) were transduced with either shTGM2-1, shTGM2-2, or shSCRMBL. Time-lapse imaging and proliferation assay were performed to determine a rescue from cell death upon TGM2 knockdown. **A** Fold change of cell number of HCT-116 p53^wt^ and HCT-116 p53^−/−^ cells upon TGM2 knockdown in comparison to shSCRMBL control determined at day three after transduction. Data are presented as mean ± SD of three independent experiments (**P < 0.01, Mann–Whitney *U* test). **B** Single cell tracking of HCT-116 p53^wt^ and HCT-116 p53^−^^/−^ cells after TGM2 knockdown with shTGM2-1 and **(C)** shTGM2-2. Cumulative cell death events are shown over time (***P < 0.001, Log-rank test). **D** Direct visualization of p53 activation upon TGM2 knockdown by time-lapse video-microscopy. Sequence of phase contrast images, tdTOMATO fluorescence of shTGM2-1 [1] and p53-driven destabilized GFP reporter [2], depicting the same field of view over the time course of 30 hours as indicated in the corresponding panels in I–VIII. The yellow circles designate tracked cells over time. (I–VIII) show corresponding sequence of fluorescence images taken at the same time points as the phase contrast images. (I) Shown are two representative HCT-116 cells. (II and III) 6-8 hours after lentiviral transduction of shTGM2-1 both HCT-116 cells express the red fluorescent tdTOMATO reporter, indicating a knockdown of TGM2. (IV-VI) Another 4–10 hours later both cells express the green fluorescent (GFP) p53 reporter, indicating the induction of p53 activity. (VII and VIII) About 24 hours after transduction both HCT-116 cells subsequently undergo apoptosis (white arrows). Movie S1 shows all assembled images (3 min temporal resolution) of the same sequence.

Next, we assessed whether TGM2 directly interferes with p53 to block p53-mediated apoptosis induction. We performed a proximity ligation assay in SW480 cells and in patient-derived CRC tumor cells. As shown in Fig. 7A and B, using this sensitive technology we indeed demonstrated a physical interaction of p53 and TGM2 by the accumulation of nuclear dots in SW480 cells as well as in primary patient cancer cells (Fig. 7C, D). The interaction was further confirmed by Co-immunoprecipitation of TGM2 and phosphorylated p53 in SW480 and HCT-116 CRC cell lines (Fig. 7E). Super-resolution microscopy [39] of TGM2 and phosphorylated p53 revealed the colocalization of TGM2 and phosphorylated p53 at single-molecule resolution mainly in the nucleus of HCT-116 and SW480 cells (Fig. 7F and Supplementary Fig. S10). Then, we evaluated the functional impact of p53 on the apoptosis induction by TGM2 knockdown. For this purpose, we utilized the CRC cell line HCT-116 p53^−/−^, which has a genetically engineered genomic deletion of p53, and the parental cell line HCT-116 p53^wt^ as a control. After shRNA-mediated TGM2 knockdown, we determined the cell expansion. Whereas HCT-116 p53^wt^ cells showed a significant reduction of cell expansion upon TGM knockdown, the HCT-116 p53^−^^/−^ cells were largely resistant to the TGM2 knockdown and equally expanded as the shSCRMBL-transduced controls (Fig. 8A). To further confirm these results, we looked continuously at single cell resolution by time-lapse-microscopy-based cell tracking. Similar to the results with SW480 cells, HCT-116 p53^wt^ cells strikingly triggered cell death with up to 90% of dead cells after 40 hours upon TGM2 knockdown, whereas HCT-116 p53^−/^^−^ were partly rescued from cell death (40% of dead cells) and the majority of cells divided within 40 hours (Fig. 8B, C). Last, we utilized a fluorescent reporter to quantify the activity of the transcription factor p53. A lentiviral construct carrying p53 transcriptional response elements (TREs) at a minimal CMV promoter driving the expression of a destabilized green fluorescent protein (GFP) upon p53 activation were transduced in HCT-116 p53^wt^ cells. By the continuous observation of the p53-driven GFP signal upon knockdown of TGM2 using time-lapse-microscopy-based cell tracking, we could clearly show a temporal order of events in single HCT-116 cells: after the expression of shTGM2-1 (red TOMATO expression), the p53-driven GFP signal started to increase at a time delay of approximately 6 hours and cells became strong GFP positive, before the cells died. (Fig. 8D, Movie S1). Immunohistochemistry of tumor sections from CRC patients demonstrated enhanced p53 phosphorylation in tumors with low TGM2 expression, while tumors with high TGM2 showed almost no staining for S15-phosphorylation of p53 (Suppl. Fig. 11). These data show a strong reverse association of TGM2 and p53 activity and indicate that the direct activation of p53 by the knockdown of TGM2 is clearly promoting the rapid pro-apoptotic fate of CRC cells.

## Discussion

Here we demonstrate TGM2 as a suitable target molecule for future therapeutic interventions in CRC. TGM2 fulfills essential functions for the survival and growth of CRC cells. TGM2 is overexpressed in tumor cells in patients with CRC and transamidation activity is increased in cancer tissue. These results warrant further confirmation in a larger patient cohort.

The role of TGM2 in cancer has been controversial, and a tumor suppressive function, as well as an oncogenic role, have been reported. Studies showed induction of cell death upon TGM2 expression [40, 41] and upon ablation of TGM2, as shown in breast [42], pancreatic [43, 44], and ovarian cancer [45]. Accordingly, in CRC both a tumor-promoting [35] as well as a tumor-suppressive function [30, 34, 46] of TGM2 have been described. The multi-facet enzymatic action of TGM2 is involved in many different cellular mechanisms, ranging from extracellular matrix organization, signaling, gene expression, migration, apoptosis, and autophagy. Therefore, it is likely, that different cell types respond differentially to alterations in distinct TGM2 functions, which are also influenced by the individual tumor microenvironment. These seemingly contradictory cellular functions attributed to TGM2 are puzzling and demonstrate the importance to consider the different enzymatic activities as well as isoforms of TGM2 in studies of its role in cancer biology.

Our results demonstrate that the transamidase activity and not the GTPase activity is required for cell survival and growth in CRC. Moreover, only full-length TGM2 isoform 1 with strong transamidase activity shows a pro-survival function. We have shown here that inhibition of TGM2 transamidase activity by genetic knockdown or knockout is sufficient to rapidly induce apoptosis in CRC cells. This provides a rationale to utilize TGM2 inhibitors that block transamidase activity in future studies.

Furthermore, we were able to show that the pro-survival function of TGM2 in CRC is dependent on the tumor suppressor p53. Here, a remaining activity of p53 appears to be enough to induce Caspase-3-dependent apoptosis. Although SW480 cells harbor a p53 mutation with reduced activity, the genetic ablation of TGM2 resulted in rapid induction of apoptosis and an almost abolished expansion of SW480 CRC cells in vitro and in vivo. We demonstrated a physical interaction of TGM2 and p53 in CRC cells with different methods. Super-resolution microscopy revealed a clear colocalization of TGM2 and phosphorylated p53 mainly in the nucleus, suggesting that DNA binding of p53-TGM2 may be involved. TGM2 expression prevented activation of p53 signaling, maintaining phosphorylated p53(S15) levels low. Thus, p53 signaling can be hindered to induce pro-apoptotic pathways such as activating Caspase-3 in CRC. In renal cell carcinoma an inverse relationship of TGM2 and p53 expression has been detected. TGM2 cross-links p53 molecules to form inactive p53 polymers by directly targeting the DNA-binding domain of p53. These p53 polymers are degraded by induced autophagy [47, 48]. Further, TGM2 binds to the same region of p53 where HDM2 binds, and acts as a chaperone to locate p53 to the autophagosome receptor p62, which is then irrespective of TGM2 crosslinking activity [48]. Therefore, HDM2 and TGM2 both regulate p53 stability in renal cell carcinoma. Whether a similar mechanism also applies in CRC requires further investigation. Another study also indicated a transamidase independent role of TGM2 in regulation of p53 signaling, demonstrating that TGM2 itself phosphorylated p53 leading to a disruption of the interaction of p53 and its regulator protein Mdm2 [49]. Recently, it was even postulated that TGM2 is located downstream of p53 and thus a direct target in p53 signaling [50]. In CRC, reduction in p53 function is a late event in the adenoma–carcinoma sequence [51, 52]. Mutations of p53 occur in approximately 50% of all CRC patients and are associated with worse survival for patients treated with chemotherapy [53]. However, while p53 mutations often lead to p53 inactivation, many mutations retain significant activity [54] or even acquire oncogenic properties and thus drive tumor formation, invasion and metastasis through gain-of-function activities or dominant negative inhibition of wild-type p53 [55]. The upregulation of TGM2 in CRC cells may serve as an escape mechanism of CRC cells to further inhibit remaining p53 even in mutated cases. TGM2 inhibition may be best suited for drug combination approaches. The reactivation of remaining p53 activity may lower the threshold for apoptosis induction by synergistic compounds.

Our data show a potent mechanism of how CRC cells can block p53-induced apoptosis without the appearance of genetic aberrations affecting the central tumor suppressor p53. Since p53 inactivation is a common mechanism in most cancers, further studies on the here described TGM2-dependent mechanism are warranted which may be of importance in other cancers beyond CRC.

## Materials and methods

Detailed descriptions regarding the experimental procedures are provided in the Supplemental Materials and Methods.

### Study approval

Written informed consent was obtained from all patients prior to inclusion in the study in accordance with the Declaration of Helsinki and local laws and regulations, and the study was approved by the institutional ethics review board (Number: SGI-04-2014).

All animal experiments were performed according to protocols approved by the state government.

### Immunohistochemistry

For immunohistochemistry, colon cancer and paired noncancerous colon tissues were obtained from 10 CRC patients undergoing surgical resection. Paraffin-embedded sections were obtained from the biobank of the Goethe University Frankfurt Cancer Center’s Tissue Procurement Facility.

Each sample was stained by using anti-TGM2 antibody (1:100, CUB7402) and anti-Ki67 antibody (1:200, SP6) (both Abcam, Cambridge, UK) and multiview IHC kit (Enzo Life Sciences, Lörrach, Germany) was used to visualize immunoreactivity according to the manufacturer’s protocol. Tissues were counterstained with hematoxylin and imaged with a microscope. All slides were reviewed independently by two investigators.

### Isolation of primary colon cancer cells

Fresh human colon cancer and adjacent normal mucosa tissues were obtained from patients undergoing surgical resection between January 2014 and December 2016 at Goethe University Hospital Frankfurt or at Bethanien-Hospital (Frankfurt, Germany), who had given informed consent. To obtain single cell suspensions, tissues were dissociated as described before [56]. Magnetic cell separation was performed to obtain an epithelial cell-enriched suspension using the human Tumor Cell Isolation Kit from Miltenyi Biotec (Bergisch Gladbach, Germany) according to the manufacturer’s instructions. Purified cells were resuspended in culture medium and the purity of the isolated cells was verified by flow cytometry.

### Protein expression analysis

Protein expression of TGM2, p53, p53(S15), p53(S46) and p53(S392) was detected by Simple Western^TM^ assays using the Wes^TM^ System following the manufacturer’s protocol (Bio-Techne, Wiesbaden, Germany). Data were generated by the application of data analysis Compass software for Simple Western instruments.

### Transglutaminase activity assay

Transamidase activity of TGM2 was assessed in isolated primary cells from fresh tumor tissue and its corresponding normal tissue from eight CRC patients as described previously. Furthermore, it was assessed in different CRC cell lines and in lentiviral manipulated cells 72 hours after transduction. TGM2 activity was determined using Tissue Transglutaminase Microassay kit (Zedira, Darmstadt, Germany) following the manufacturer’s instructions.

### Cell lines and cell culture

The human colorectal cancer cell lines SW480 and HCT-116 were obtained from CLS Cell Lines Service GmbH (Eppelheim, Germany). The p53 knockout colon cancer cell line HCT-116 (p53^−/−^) was obtained from Accegen Biotechnology (Köln, Germany).

### Generation of shRNA constructs

For the knockdown of TGM2, two shRNAs targeting TGM2 (shTGM2-1; 5′CCGGTATCACCCACACCTACAAATACTCGAGTATTTGTAGGTGTGGGTGATATTTTG-3′, and shTGM2-2; 5′CCGGTTGTGCTGGGCCACTTCATTTCTCGAGAAATGAAGTGGCCCAGCACAATTTTTG-3′) were constructed and cloned into a third generation self-inactivating HIV-1 based lentiviral vector system on the backbone of pLKO.1. Lentiviral packaging was carried out as described previously [57, 58]. Transcription efficiency was monitored by detecting red fluorescent protein (tdTOMATO) via flow cytometry.

### Generation of CRISPR constructs

pLentiCRISPRv2 vectors containing the different sgRNAs complementary to an intron-exon region located to exon 1 or exon 5 of TGM2 (5′-CACCGTGATACTCACCCTCGGCCA-3′; or 5′-CACCGCTCTGACACAGTTTGAAGA-3′), obtained by target-specific oligonucleotide annealing using the GoldenGate protocol. The puromycin resistance cassette in pLentiCRISPRv2 was replaced by blue fluorescent protein (eBFP2). A pLentiCRISPRv2 vector containing a non-targeting sgRNA was used as control.

### Generation of TGM2 isoforms and mutant constructs

Five different TGM2 constructs were used in this study. Full-length or short TGM2 constructs, as well as enzymatic TGM2 mutants have been described previously [31]. The coding sequence of human TGM2-isoform 1 and -isoform 2 were cloned into the lentiviral expression vector pRRL.PPT.SFFV.IRES.VENUSnucmem, that coexpresses a nuclear membrane-bound fluorescent protein VENUS [58]. Point mutations were inserted into the coding sequence of human TGM2-isoform 1, resulting in TGM2 constructs lacking either transamidase activity (TGM2^C277S^), GTP binding activity (TGM2^R580A^), or both (TGM2^C277S+R580A^). All constructs were verified by Sanger sequencing. The parental vector pRRL.PPT.SFFV.IRES.VENUSnucmem was used as vector control.

### Usage of a lentiviral p53 activity reporter construct

The pGF-p53-mCMV-EF1α-Puro lentiviral vector (System Biosciences, USA) co-expresses a destabilized copepod GFP (dscGFP; 2-hours half-life) and luciferase from p53 transcriptional response elements (TREs) paired with a minimal CMV promoter (mCMV). The mCMV promoter alone delivers negligible expression, but when downstream of p53 TREs, drives expression of dscGFP in response to p53 activity, in order to quantitatively measure p53 activity at p53 TREs by fluorescence.

### In vivo xenograft experiments

Animals were maintained under specific pathogen-free conditions. NOD.CB17-*Prkdc*^*scid*^ (NOD-SCID) mice were purchased from Jackson Laboratory (Sacramento, California, USA) or bred at the Animal Facility of the Georg-Speyer-Haus, Frankfurt. Transplantation experiments were performed as previously reported [56]. Female mice were used for experiments at 6–8 weeks of age. SW480 cells were transduced with either shSCRMBL, shTGM2-1, or shTGM2-2. 72 hours after transduction cells were harvested, resuspended 1:1 in Matrigel (BD, Heidelberg, Germany) and culture medium and were injected subcutaneously into the flank at 5 × 10^4^ cells per mouse. Eight mice were allocated per group. Tumor growth was measured twice weekly using a caliper. Mice were sacrificed when tumor size reached a diameter of 1.0 cm. The investigator was not blinded concerning group allocation.

### Time-lapse imaging and single cell tracking

Long-term time-lapse imaging and single-cell tracking were done as described previously by Rieger et al. [37]. Single-cell tracking was performed by scientists using a self-written computer program (TTT).

### RNA sequencing

100 000 SW480 cells or 50 000 HCT-116 cells were transduced for 48 hours or 24 hours, respectively, with either shTGM2-1 or shSCRMBL as a control. Transduction efficiency was validated by FACS. RNA isolation was performed using RNAeasy Isolation kit (Qiagen, Hilden, Germany) according to the manufacturer’s instructions. The quality and concentration of libraries were determined by Agilent 2100 Bioanalyzer and RiboGreen fluorescence on Qubit (Thermo Fisher Scientific, Waltham, Massachusetts, USA). The library for SW480 cells was prepared using Illumina TruSeq Stranded Total RNA Library Prep kit and sequenced on an Illumina HiSeq 4000 System (Illumina, San Diego, California, USA) using a 100-bp paired-end approach. The resulting FASTQ files were aligned to the hg19 draft of the human genome and counts per gene were estimated using STAR (v.2.5.3). Read count normalization, differential expression calculation, and multidimensional scaling were performed using the DESeq2 package in R (v.3.4.1). Pathway analysis was performed using the gene set enrichment analysis (GSEA) software (v.3.0) on the hallmark gene sets (H) dataset. Only pathways with an FDR-corrected q-value below 25% were considered significant. Top 16 hits were shown. The library preparation and RNA-seq data analysis of HCT-116 cells are described in the supplementary Materials and Methods. The raw data is provided in Gene Expression Omnibus under the accession number GSE130482.

### Proteome Profiler Human Apoptosis Array

SW480 cells were transduced with either shSCRMBL as a control, with shTGM2-1 or shTGM2-2. Three days after transduction, cells were lysed on ice in M-PER Mammalian extraction Reagent (Thermo Fisher Scientific). The Proteome Profiler Human Apoptosis Array kit (R&D Systems, Abingdon, UK) procedure was performed as described in the manufacturer’s instructions. Quantification was performed using ImageJ Software.

### Proximity ligation assay

Proximity ligation assay was performed using the Duolink in situ Starter kit (mouse/rabbit) from Merck (Darmstadt, Germany) according to the manufacturer’s instructions. The primary antibodies and dilutions were as follows: mouse anti-TGM2 antibody (CUB7402, Abcam) at 1:100 and rabbit anti-p53 (S15) antibody (D4S1H, Cell Signaling Technology, London, UK) at 1:100. Finally, the slides were analyzed using a fluorescence microscope.

### Co-immunoprecipitation

Immunoprecipitation was performed using Dynabeads^TM^ Protein G Immunoprecipitation kit (Thermo Fisher Scientific) according to the manufacturer’s instructions. Briefly, whole cell lysates were extracted using Pierce^TM^ IP lysis buffer containing protease inhibitor. The antibody magnetic bead complex was prepared by adding 5 µg of mouse anti-TGM2 antibody (CUB7402, Abcam) to the magnetic beads. Antigen was immunoprecipitated by adding the protein extracts to the magnetic antibody complex for one hour and eluted at 70 °C for 10 minutes. The eluted target protein was subjected to Protein Simple capillary Simple Western technology (WES) for protein detection of TGM2, p53, p53(S15), p53(S46), and p53(S392).

### Super-resolution microscopy

Super-resolution microscopy of HCT-116 and SW480 cells immunostained for TGM2 and p53(S15) was conducted using the DNA-PAINT principle following experimental procedures that were previously published [59, 60].

### Statistical analysis

All statistical analyses were performed in GraphPad Prism 6. All values are presented as means ± SD from at least three independent experiments, unless otherwise stated. Independent *t*-test (two-tailed, unpaired, equal variances) was performed for comparison of data unless otherwise stated. The significance level for all tests was set to *α* = 5%. *, *P* value < 0.05; **, *P* value < 0.01; ***, *P* value < 0.001.

## Supplementary information

Supplemental Material

Supplemental Table S2

Movie S1
