# Supplementary material for: Transglutaminase 2 promotes tumorigenicity of colon cancer cells by inactivation of the tumor suppressor p53
Source: Oncogene. 2021 Jun 8;40(25):4352–67. doi: 10.1038/s41388-021-01847-w (PMC8225513; doi:10.1038/s41388-021-01847-w)
Supplement: Supplementary file 1 — Supplemental Material [file 41388_2021_1847_MOESM1_ESM.pdf]

Supplementary Table S1

Table S1: Patient and tumor characteristics								
**Patient**	**Age (years)**	**Tumor location**	**T-stage**	**N-stage**	**M-stage**	**UICC stage**	**Grade**	**TGM2 score**
**1**	83	Sigmoid	3	0	0	2	2	3
**2**	71	Sigmoid	3	2	0	3	2	4
**3**	78	Ascending colon	2	0	0	1	2	1
**4**	69	Sigmoid	3	1	0	3	2	5
**5**	48	Ascending colon	2	1	1	4	3	5
**6**	58	Sigmoid	2	0	0	1	2	2
**7**	80	Descending colon	2	0	0	1	2	1
**8**	77	Descending colon	3	1	1	4	2	5
**9**	61	Cecum	2	0	0	1	2	2
**10**	87	Ascending colon	3	0	0	2	2	3

Abbreviation: UICC, International Union Against Cancer; TGM2 score, Transglutaminase 2 staining score; Grade, tumor grading

Supplementary Figures

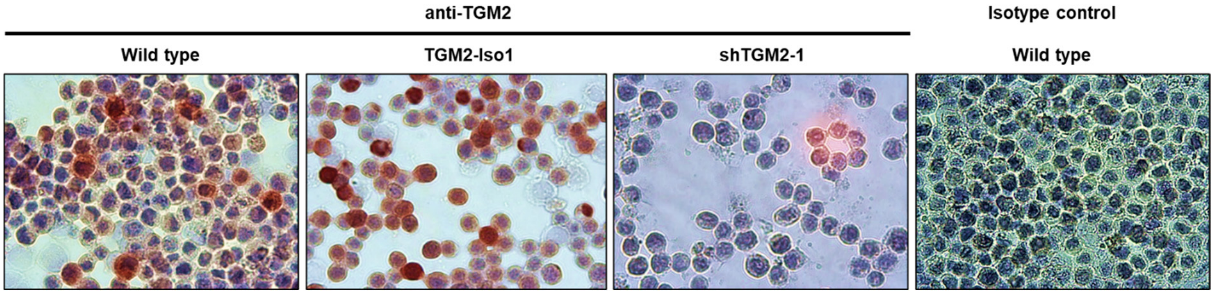

Figure S1. Confirmation of antibody specificity against TGM2. Representative microphotographs of SW480 cells after immunohistochemical (IHC) stainings of TGM2 (brown) or isotype control. Wild type SW480, SW480 cells after lentiviral overexpression of full-length TGM2-Isoform 1 (TMG2-Iso1) and after lentiviral TGM2-knockdown (shTGM2-1) were stained to show specificity of the anti-TGM2 antibody used for IHC.

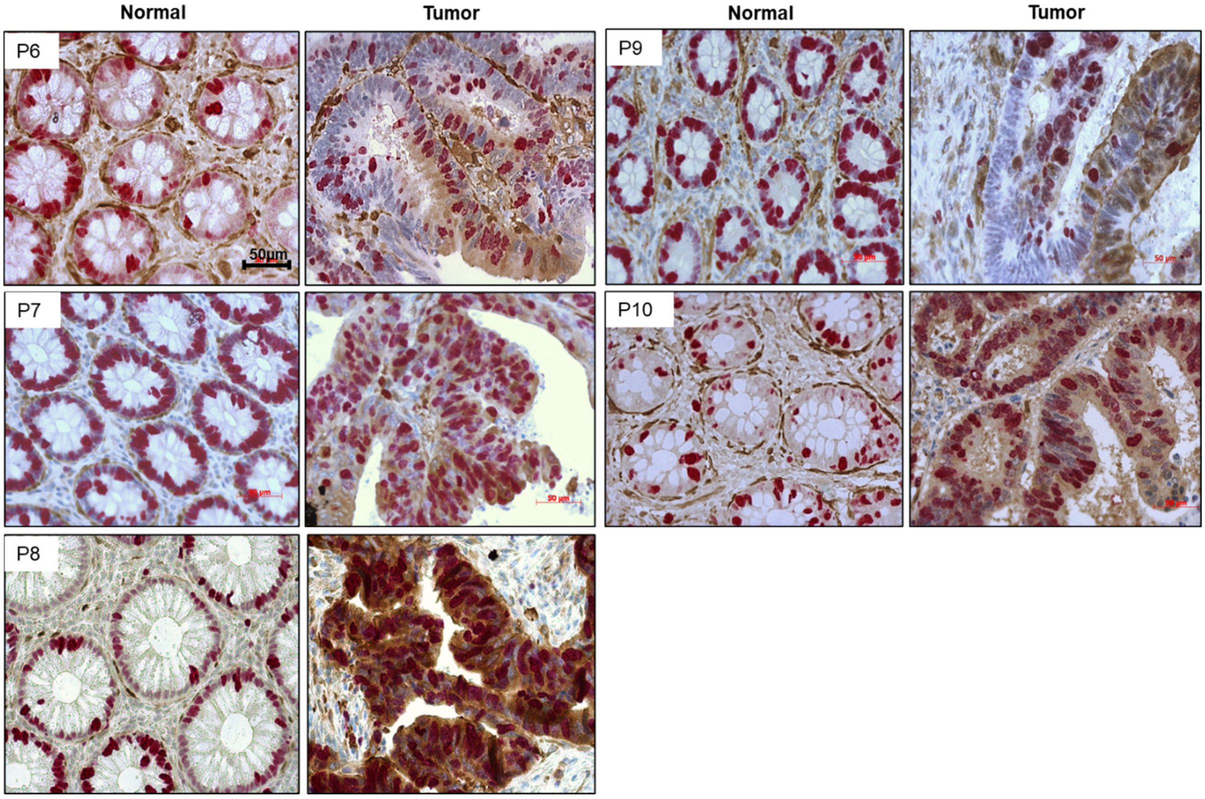

Figure S2. TGM2 protein expression in matched tumor/normal colon tissue of CRC patients. Representative microphotographs of immunohistochemical stainings of TGM2 (brown) and Ki67 (red) in paired tumor and corresponding normal tissue from ten colorectal cancer patients (P6-10, P1-5 are shown in Fig. 1A). Scale bar, 50 µm.

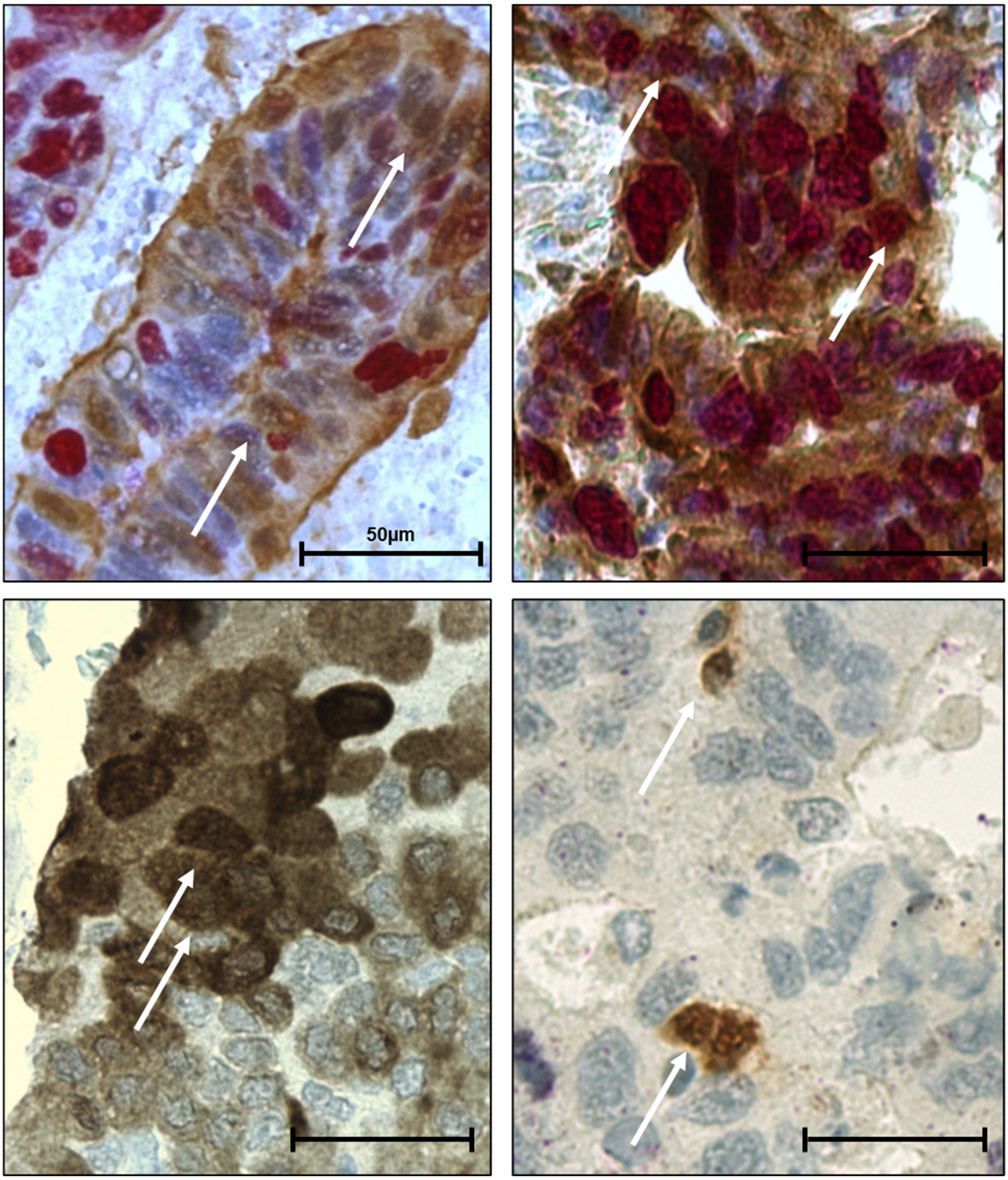

Figure S3. Nuclear and cytoplasmic localization of TGM2 in CRC tissue. Representative microphotographs of immunohistochemical stainings of TGM2 (brown) and Ki67 (red) in tumor tissue from colorectal cancer patients showing nuclear localization (arrows) and cytoplasmatic staining of TGM2. Scale bar 50µm.

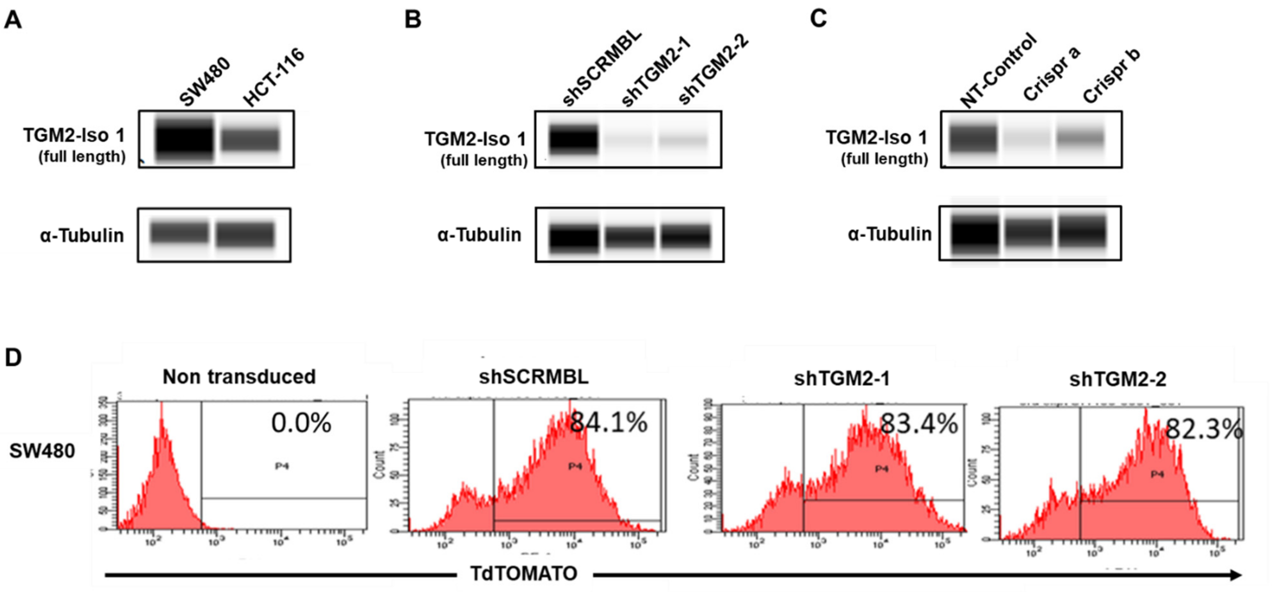

Figure S4. Analysis of TGM2 protein expression in colon cancer cell lines. (A-C) Representative protein expression data of TGM2-isoform 1 expression in colon cancer cell lines, and after knock-down or knock-out of TGM2. α-Tubulin served as loading control. (A) TGM2-isoform 1 expression in SW480 and HCT-116 cells. (B) TGM2-isoform 1 expression in SW480 cells after transduction with TGM2 knock-down constructs (shTGM2-1, shTGM2-2) or control vector (shSCRMBL). (C) TGM2-isoform 1 expression in SW480 cells after lentiviral transduction with TGM2 knock-out constructs (CRISPR a, CRISPR b, or control vector. (D) Representative FACS plots after lentiviral transduction of shRNA constructs showing high transduction efficiency.

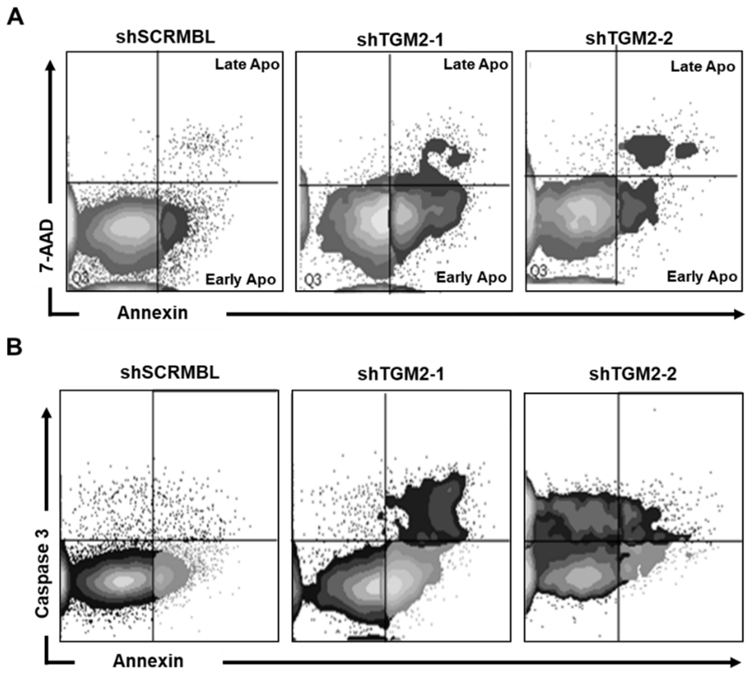

Figure S5. Representative FACS plots of Annexin and Caspase-3 assays after TGM2 knock-down. SW480 cells were transduced with shTGM2-1, shTGM2-2 or shSCRMBL. Three days after transduction, apoptosis was determined based on Annexin V/7-AAD and (B) Caspase-3/Annexin V staining. The quantification is shown in Figure 4B and 4C. (Apo, Apoptosis).

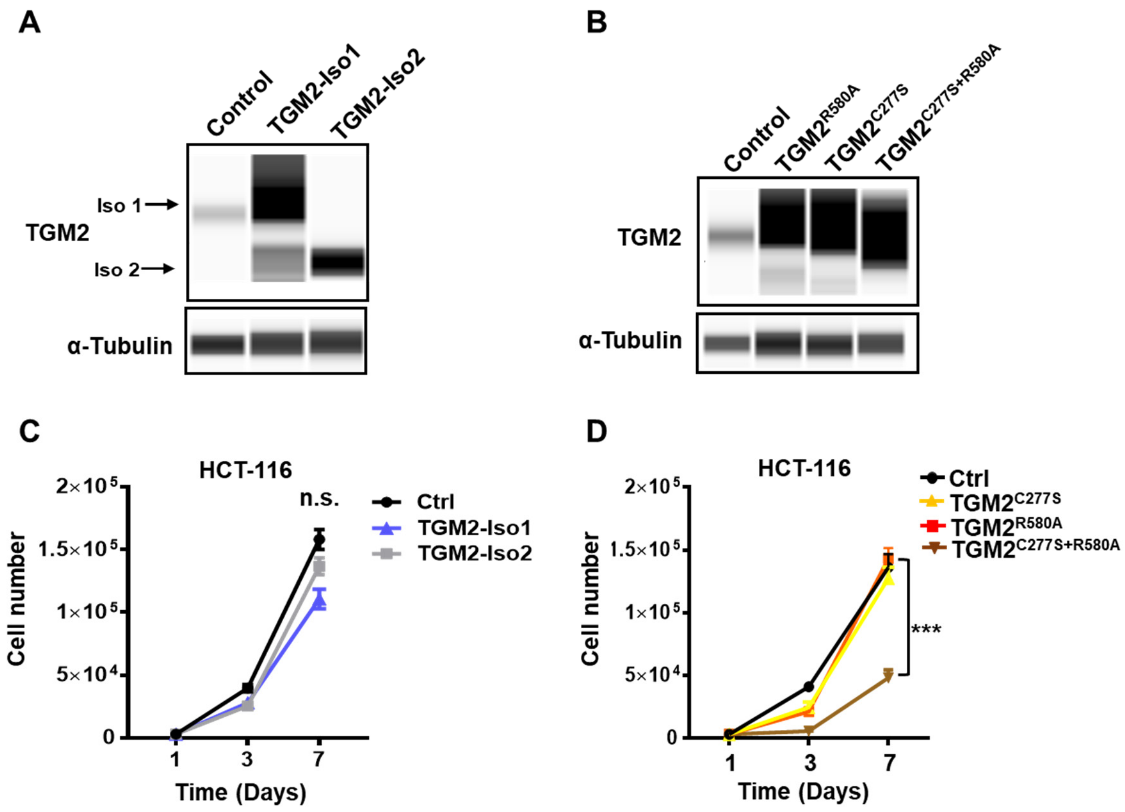

Figure S6. Gain-of-function studies with TGM2 isoforms and TGM2 mutant constructs in CRC cell lines. (A) Representative protein expression data showing TGM2 expression in SW480 cells after lentiviral overexpression of TGM2-isoform 1 or 2 and (B) in SW480 cells after lentiviral overexpression of TGM2 mutant constructs, TGM2^{C277S}, TGM2^{R580A}, TGM$^{C277S+R580A}$, or vector control. α-Tubulin served as loading control. (C) Cell expansion of HCT-116 cells overexpressing TGM2-isoform 1 or 2 in comparison to vector control transduced cells (Ctrl). (D) Cell expansion of HCT-116 cells overexpressing either TGM2^{C277S}, TGM2^{R580A}, TGM$^{C277S+R580A}$, or vector control (Ctrl). Cells were counted at day 3 and 7 after transduction. Results are presented as mean ± SD of three independent experiments. Mann-Whitney U test; *** P<0.001.

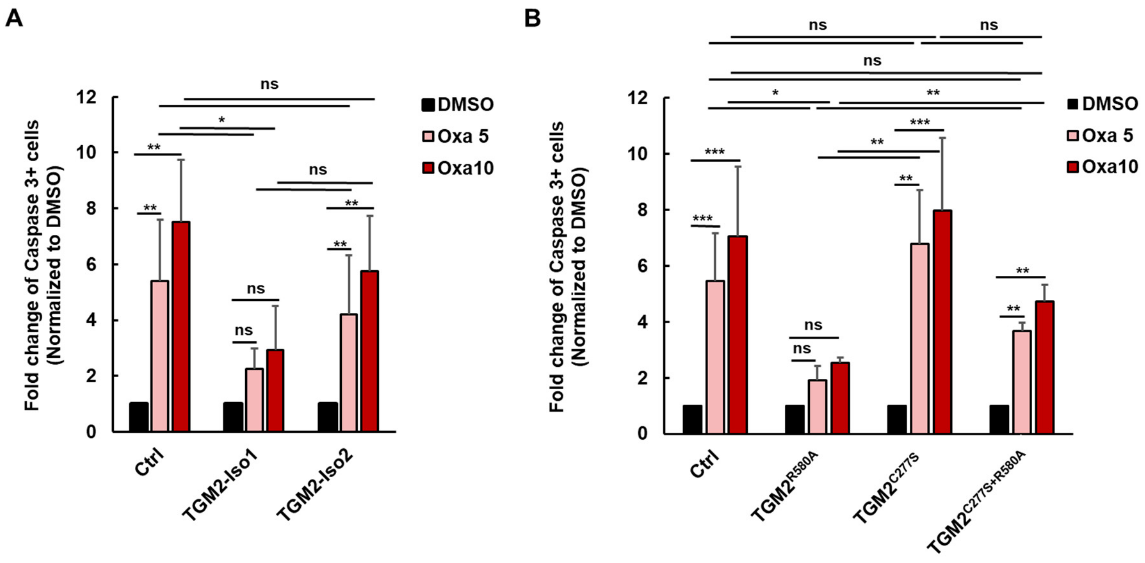

Figure S7. Overexpression of TGM2-isoform 1 and the active transamidase mutant TGM2^{R580A} prevents Caspase-3 activation by oxaliplatin. (A) SW480 cells overexpressing TGM2-isoform 1, TGM2-isoform 2 or control vector (Ctrl) as well as (B) SW480 cells overexpressing mutant forms TGM2^{R580A}, TGM2^{C277S}, TGM2$^{C277S+R580A}$ or control vector (Ctrl) were treated with Oxaliplatin (Oxa) for 72 hours and Caspase-3 assay was performed. Shown is the induction of Caspase-3 positive cells normalized to DMSO treated cells. A comparison of Caspase 3 activation in control vector transduced cells and cells expressing TGM2-isoforms and TGM2 mutant constructs was conducted. Data are presented as mean ± SD of three independent experiments. Mann-Whitney U test; * $P<0.05$; ** $P<0.01$, *** $P<0.001$.

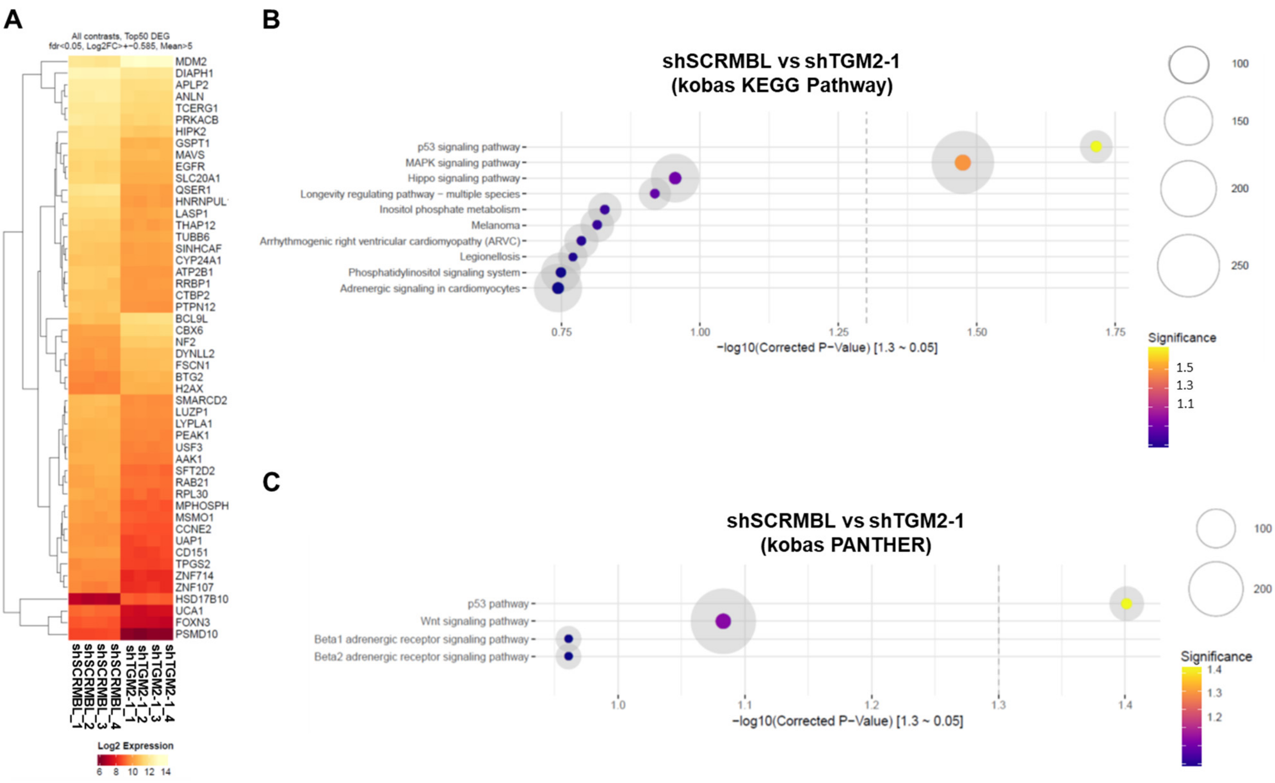

Figure S8. Gene expression profiling by RNA-seq of HCT-116 cells after transduction with either shTGM2-1 or shSCRMBL. (A) Unsupervised hierarchical clustering of the top 50 differentially expressed genes (DEGs) upon TGM2 knock-down across the four biological replicates. List of regulated genes is presented in Supplementary Table S2. (B) Scatter plot of gene set enrichment analysis of DEGs showing the top regulated pathways by KEGG Pathway or (C) PANTHER analysis (Corrected P-value<0.02, Top 50 Sets). The color and size of each dot represent the Rich factor and the number of DEGs mapped to the indicated pathway, respectively.

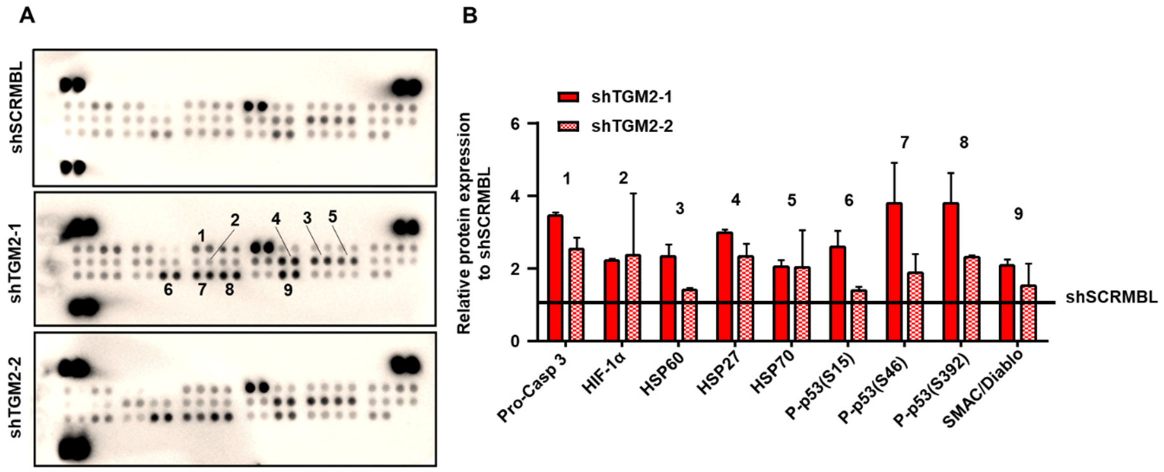

Figure S9. Human Proteome Profiler Apoptosis Array. (A) Representative result of the Proteome Profiler Array™-Human Apoptosis Array with lysates from SW480 cells 72 hours after lentiviral transduction with either shTGM2-1, shTGM2-2 or shSCRMBL. (B) Quantitative densitometrical protein expression analysis of the results. Changes of protein expression upon TGM2 knock-down were normalized to shSCRMBL control (n = 2). 2-fold enriched proteins are shown. Numbers indicate corresponding protein spots on the array. The data is represented as mean + SD; (P, Phosphorylated).

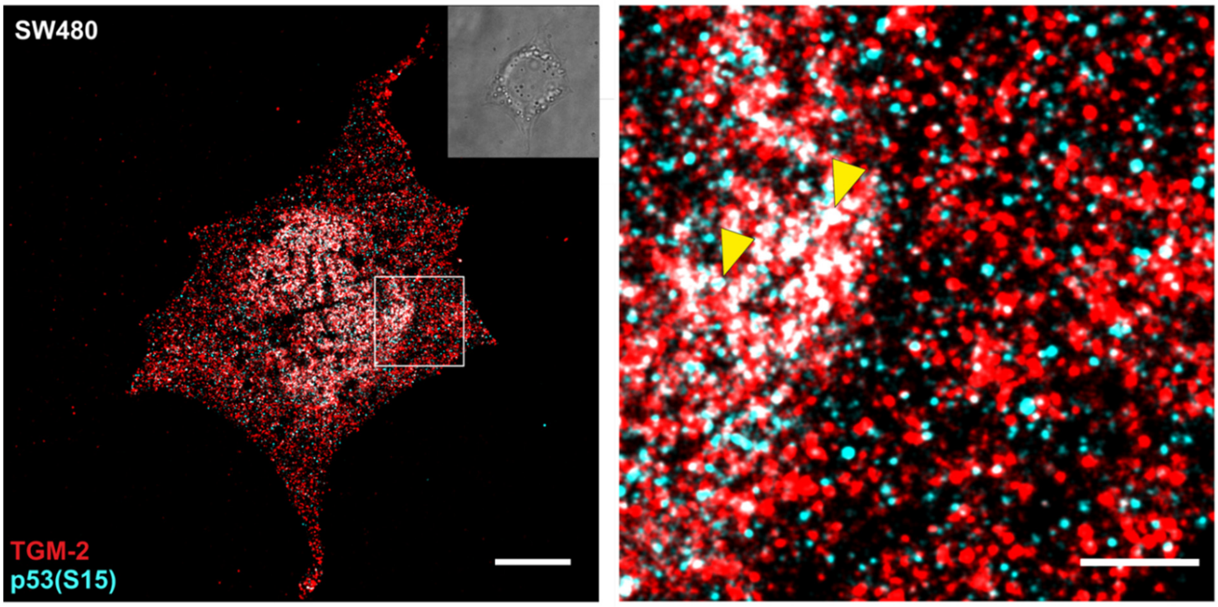

Figure S10.**TGM2 and p53(S15) co-localization in SW480 cells detected via DNA-PAINT super-resolution microscopy.** A super-resolved image is obtained from binding events of short, fluorescently labeled DNA oligonucleotides to DNA-labeled antibodies. DNA-PAINT image (left) of TGM2 (red) and phosphorylated p53(S15) (cyan) immunostained with secondary antibodies carrying P1 and P5 DNA docking strands and labeled with complementary DNA imager strands labeled with ATTO 655. Zoom-in of region marked in the left image is shown (right). White regions in the super-resolved images show overlapping signals of TGM2 and p53(S15) and thus indicate an interaction of both proteins. Some co-localization events are highlighted with yellow arrow heads. Scale bars represent 5 µm (left) and 1 µm (right).

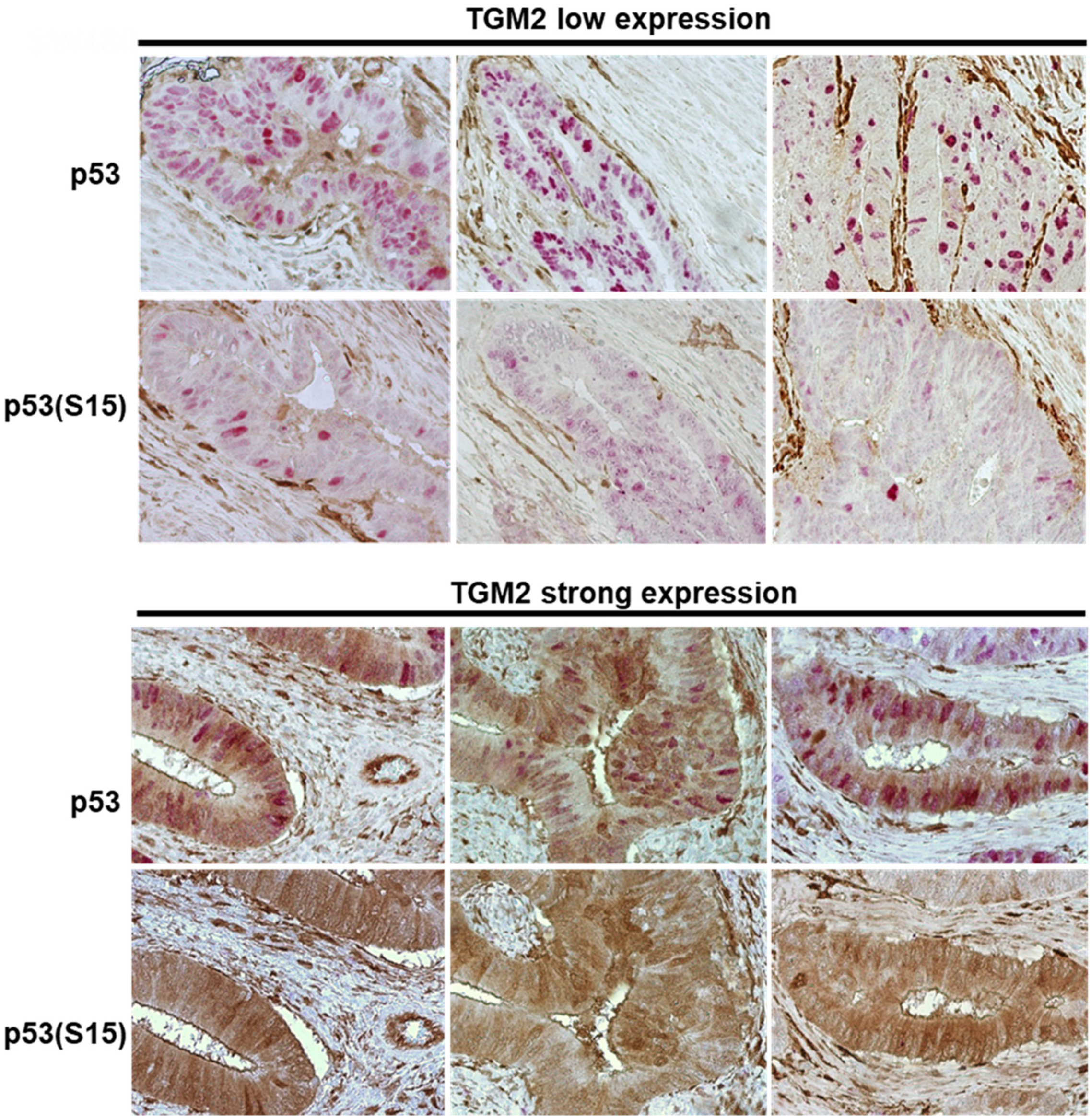

Figure S11. Enhanced p53 phosphorylation in CRC patients with low TGM2 expression. Representative microphotographs (magnification 200x) of immunohistochemical (IHC) stainings of TGM2 (brown) and p53 (red) or phosphorylated p53(S15) (red) in tumor tissue from CRC patients with low or strong expression of TGM2.

Supplementary Materials and Methods

Immunohistochemistry

For immunohistochemistry, colon cancer tissue and paired noncancerous colon tissues were obtained from 10 CRC patients undergoing surgical resection of the primary tumor. Samples were obtained from the biobank of the Goethe University Frankfurt Cancer Center's Tissue Procurement Facility. The use of the samples for research purposes was approved by the Ethics Committee of the Goethe University Frankfurt and all patients gave written consent before operation.

Each sample was deparaffinized by 2 changes of xylol, followed by series of descending grade of alcohol and equilibrated in PBS. Antigen retrieval was performed by Aptum 2100-Retriever using One R-Universal buffer (Aptum Biologics Ltd., Southampton, UK) for 30 minutes. Subsequently sections were blocked for 10 minutes and incubated with mouse anti-human TGM2 antibody (1:100, CUB7402) and rabbit anti-human Ki67 antibody (1:200, SP6) (both Abcam, Cambridge, UK), rabbit anti-human p53 antibody (1:100, #9282, Cell Signaling Technology, Danvers, Massachusetts, USA) or rabbit anti-phospho-p53(S15) (1:100, #AF1043, R&D Systems, Minneapolis, Minnesota, USA) for 1 hour. Multiview (mouse-HRP/rabbit-AP) IHC kit (Enzo Life Sciences, Lörrach, Germany) was used to visualize immunoreactivity according to the manufacturer's protocol. TGM2 was visualized using DAB (3,3'-Diaminobenzidine) chromogen (brown) while Ki67, p53 or phosphorylated p53(S15) was visualized using AP (Alkaline phosphatase) chromogen (red). Tissues were counterstained with hematoxylin to obtain nuclear staining. All slides were reviewed independently by two investigators. Immunoreactivity was scored by assessing the percentage of positive TGM2 stained cells. TGM2 intensity in epithelial mucosa or tumor cell cytoplasm was classified into a five-level staining score: 0= no staining, 1= weak staining with <10% TGM2-positive tumor cells, 2= weak-moderate staining with 10-30% TGM2-positive tumor cells, 3= moderate

staining 30-70% TGM2-positive tumor cells, 4= strong staining with 70-90% TGM2-positive tumor cells and 5= very strong staining with > 90% TGM2-positive tumor cells.

To confirm the specificity of the monoclonal TGM2 antibody we stained wild type SW480 cancer cells as well as TGM2 overexpressing and TGM2 knock-down SW480 cells. There was a strong staining in TGM2 overexpressing cells, while CRC cells after TGM2 knock-down showed no staining (Supplementary Fig. S1). Further, an isotype control was used to exclude unspecific antibody binding.

Isolation of primary cells from patient specimens

Fresh human colon cancer and adjacent normal mucosa tissue were obtained from patients undergoing surgical resection at Goethe University Hospital Frankfurt or at Bethanien-Hospital (Frankfurt, Germany), who had given informed consent. All tissues were collected under protocols approved by the ethics committee of the University Hospital Frankfurt. All samples were characterized by a pathologist. To obtain single cell suspension, solid tissues were minced into small fragments, washed with PBS containing penicillin/streptomycin followed by enzymatic dissociation with 200 U/ml Collagenase type III, 100 U/ml Dispase and 100 U/ml DNase I (all Worthington Biochemical Corp., Lakewood, NJ, USA) in HBSS for 45 minutes at 37°C. During enzymatic incubation, cell suspension was subjected to MACS tissue-dissociator under a defined program every 15 minutes. The digested material was filtered through a sterile 40 µm-nylon mesh (BD Becton Dickinson, Heidelberg, Germany). Contaminating red blood cells were removed by osmotic lysis. Magnetic cell separation was performed to obtain an epithelial cell-enriched suspension using the human Tumor Cell Isolation Kit from Miltenyi Biotec (Bergisch-Gladbach, Germany) according to the manufacturer‘s instructions. After magnetic sorting, the viability was assessed using trypan blue exclusion and purified cells were resuspended in serum-free DMEM/F12 (Gibco, Thermo Fisher Scientific Waltham,

Massachusetts, USA) supplemented with 20 ng/ml epidermal growth factor and fibroblast growth factor, 2 % N2 supplement (Thermo Fisher Scientific, Waltham, Massachusetts, USA), 20 mM HEPES, and 50 U/ml penicillin/streptomycin. The purity of the isolated cells was verified by flow cytometry.

Protein expression analysis by Simple Western technology

For protein extraction, cells were lysed on ice in M-PER Mammalian extraction Reagent supplemented with Pierce Protease and Phosphatase Inhibitor Mini Tablets (Thermo Fisher Scientific) to prevent proteolytic degradation of the protein. Protein was quantified using Pierce™ Coomassie plus (Bradford) Protein Assay kit (Thermo Fisher Scientific). Protein expression was detected by Simple Western™ assays using the Wes™ System following the manufacturer's protocol (Bio-Techne, Wiesbaden, Germany). The system combines capillary electrophoresis for protein separation, immobilization and immunodetection techniques. 50-400 ng of protein was loaded in each well. An optimized defined run setting was used, except for phosphoproteins wherein primary antibody time was readjusted. The primary antibody dilution was 1:50 to 1:20. The following primary antibodies were used: anti-TGM2 (CUB7402, Abcam), anti-p53 (1C12,), anti-phospho-p53(S15) (16G8, both Cell Signaling Technology), anti-phospho-p53(S46) (#AF1489) and anti-phospho-p53(S392) (#AF2996, both R&D Systems). Protein expression was validated using High-Dynamic-Range (HDR) multi-image analysis for every tested sample. Alpha-Tubulin (DM1A, Cell Signaling Technology) served as loading control. Quantification of chemiluminescence was based on peak area after correction for a baseline signal. Data were generated by the application of data analysis Compass software for Simple Western instruments.

Transglutaminase activity assay

Transamidase activity of TGM2 was assessed in isolated primary cells from fresh tumor tissue and its corresponding normal tissue from eight CRC patients as described previously. Furthermore, it was also assessed in different CRC cell lines and in lentiviral manipulated cells 72 hours after transduction. TGM2 activity was determined using Tissue Transglutaminase Microassay kit (Zedira, Darmstadt, Germany) following the manufacturer's instructions. Briefly, cells were lysed on ice in M-PER Mammalian extraction Reagent (Thermo Fisher Scientific) and protein concentration was determined subsequently. Protein samples were incubated with reaction buffer containing calcium, DTT and biotin-pepT26 in the wells of the microtiter plate. For negative control, EDTA was added. Each well has been covalently coupled to spermine. In the presence of active TGM2, spermine is incorporated into the y-carboxamide of the glutaminyl residue of biotin-pepT26. Enzymatic reaction is determined by its interaction with streptavidin labelled peroxidase. A substrate solution for peroxidase was added for color development. The color intensity was measured on the Infinite 200 microplate reader (Tecan Group, Männedorf, Switzerland).

Cell lines and cell culture

The human colorectal cancer cell lines SW480 and HCT-116 were obtained from CLS Cell Lines Service GmbH (Eppelheim, Germany). The p53 knock-out colon cancer cell line HCT-116 ($p53^{-/-}$) was obtained from Accegen Biotechnology (Köln, Germany). Cells were cultured in McCoys medium (GibcoThermo Fisher Scientific) containing 10% fetal calf serum (FCS), 200 mM Hepes, 2 mmol L-glutamine, 50 units/ml penicillin and 50 µg/ml streptomycin, in 37°C humidified atmosphere with 5% CO2. Authentication of SW480 and HCT-116 cell lines was performed by short tandem repeat (STR) genotyping (CLS Cell Lines Service). All cell lines were mycoplasma free during the course of the experiment. All experiments were performed using cell lines which had been passaged <25 times.

Generation of shRNA constructs

For the knock-down of TGM2, a third generation self-inactivating HIV-1 based lentiviral vector system on the backbone of pLKO.1, in which the puromycin resistance cassette was replaced by an open reading frame for a nuclear membrane bound TdTOMATO (TdTOMATOnucmem), were generated. Hairpin oligonucleotides for shRNA cloning were designed and synthetically produced by Thermo Fisher Scientific. Forward and reverse oligonucleotides were annealed and ligated with their overlapping 5' ends into the lentiviral vector. Two shRNAs targeting TGM2 (shTGM2-1;5'-CCGGTATCACCCACACCTACAAATACTCGAGTATTTGTAGGT GTGGGTGATATTTTTG-3‘, and shTGM2-2; 5‘-CCGGTTGTGCTGGGCCACTTCATTTC TCGAGAAATGAAGTGGCCCAGCACAATTTTTG-3‘) were constructed.

Lentiviral production

Vesicular Stomatitis Virus-G (VSVG)-pseudotyped lentiviral particles were produced in a split genome approach by calcium-phosphate-mediated transient transfection of human embryonic kidney HEK293T producer cells. After 48 hours, supernatant was collected, filtered (45µm), and enriched by ultracentrifugation (50 000g, 2 hours). Viral titers were determined by transduction of NIH3T3 cells with different concentrations of virus supernatant.

Lentiviral transduction

CRC cells (SW480 or HCT-116) were infected with lentivirus in complete medium at a multiplicity of infection (MOI) of 5. Cells were harvested for subsequent assays 72 hours after transduction unless otherwise stated. Transduction efficiency was analyzed via flow cytometry by determining the percentage of cells positive for the reporter fluorescent protein encoded by the used construct.

Cell proliferation

Cell proliferation was determined by cell counting and MTT-assay (Roche AG, Basel, Switzerland). Cell numbers were counted in Neubauer chamber under microscope with Trypan blue exclusion. The 3-(4,5-dimethylthiazol-2yl)-2,5-diphenyltetrazolium bromide (MTT)-assay was performed according to the manufacturer's instruction in pentaplicate. The absorbance was measured on the Infinite 200 microplate reader (Tecan).

Sphere formation assay

SW480 or HCT-116 cells were suspended in serum-free DMEM/F12 medium (Gibco, Thermo Fisher Scientific) supplemented with 20 ng/ml EGF and FGF, 2% N2 supplement (Life Technologies, Carlsbad, California, USA), 20 mmol/l HEPES and 50 U/ml penicillin/streptomycin at a density of 5 000 cells per well in ultralow-attachment 24-well plates (Corning, Corning, New York, USA). Plates were scored microscopically after 7 and 14 days using Axio Observer Z-1 microscope (Carl Zeiss, Oberkochen, Germany).

Cell death Annexin V assay

Cell death was analysed with flow cytometry using Annexin V/7-AAD staining (BD Becton Dickinson) according to the manufacturer's instructions. The cells were analyzed on a FACSCanto II (BD Becton Dickinson).

Caspase-3 activity assay

Caspase-3 activity was measured using a Caspase-3 (active) Staining Kit from Abcam as described in the manufacturer's instructions. Briefly, SW480 cells were lentivirally transduced with either shSCRMBL, shTGM2-1 or shTMG2-2. 72 hours after transduction, cells were harvested and incubated with the Caspase-3 inhibitor DEVD-FMK conjugated to FITC serving as the fluorescent in situ marker. The inhibitor is internalized and binds to activated Capsase-3

in living cells. A costaining with Annnexin V and 7-AAD was performed. The cells were analyzed on a FACSCanto II.

To determine the influence of TGM2 overexpression on Caspase-3 activation, SW480 cells were transduced with TGM2 overexpression constructs (TGM2-Iso 1, TGM2-Iso 2 or control) or enzymatic mutant constructs (TGM2^{R580A}, TGM2^{C277S} or TGM2$^{C277S+R580A}$) and subsequently treated with Oxaliplatin (5 and 10 µM) (Tocris Bioscience, Bristol, UK). After 3 days of treatment Caspase-3 activity was measured as described before.

Overexpression of TGM2 variants after endogenous TGM2 knock-out (rescue)

SW480 cells were transduced with either TGM2 overexpressing constructs (TGM2-Iso 1, TGM2-Iso 2), TGM2 mutant constructs (TGM2^{C277S}, TGM2^{R580A}, TGM2$^{C277S+R580A}$) or control. After 3 days of transduction, cells were double-transduced with CRISPR-Cas9 constructs or controls to achieve a knock-out of endogenous TGM2. After 3, 7 and 14 days, cell proliferation and transduction efficiency were determined as described before.

Time-lapse imaging and single cell tracking

Long-term time-lapse imaging and single-cell tracking was done as described previously by Rieger *et al.* (37). In brief, SW480 cells or HCT-116 were transduced with either shSCRMBL, shTGM2-1 or shTMG2-2 to achieve a TGM2 knock-down. 20 000 cells were seeded per position in 24-well plates (Thermo Fisher Scientific) and virus particles were added with a MOI of 5 in 500 µl culture medium. Live-cell imaging was performed at a constant temperature of 37°C from day 0 to day 3. Phase contrast pictures were acquired every 2 minutes and fluorescence images every 1 hour. Time-lapse microscopy was performed with a ZeissCellobserver (Zeiss, Germany) using a 10x phase contrast objective and an AxioCamHRm camera with a self-written VBA module remote controlling Zeiss Axio Vision 4.8 software. Fluorescence was detected with HXP illumination (Osram) and the Cy3 filter set (F46-004,

AHF Analysentechnik, Tübingen, Germany) for tdTOMATO and the EGFP filter set (F46-002, AHF Analysentechnik) for destabilized cGFP. Single-cell tracking was performed by scientists using a self-written computer program (TTT). The tracked cell pedigrees served to determine time of cell division, cell death and gain or loss of fluorescent reporter expression. Dead cells are easily depicted by their shrunk, nonrefracting appearance with immobility. All cell tracking was done by scientists; the current analysis does not rely on data generated by an unsupervised computer algorithm for automated tracking.

RNA sequencing

100 000 SW480 cells or 50 000 HCT-116 cells were transduced for 48 hours or 24 hours, respectively, with either shTGM2-1 or shSCRMBL as a control. Transduction efficiency was validated by FACS. RNA isolation was performed using RNAeasy Isolation kit (Qiagen, Hilden, Germany) according to the manufacturer's instructions.

For the libraries with HCT-116 cells, 1µg of total RNA was used as input for SMARTer Stranded Total RNA Sample Prep Kit - HI Mammalian (Clontech). Total RNA and library integrity were verified on LabChip Gx Touch 24 (Perkin Elmer). Sequencing was performed on the NextSeq2000 instrument (Illumina) using P2 flowcell with v3 chemistry, resulting in average of 40M reads per library with 2x100bp paired-read setup.

The resulting raw reads were assessed for quality, adapter content and duplication rates with FastQC (Andrews S. 2010, FastQC: a quality control tool for high throughput sequence data. Available online at: http://www.bioinformatics.babraham.ac.uk/projects/fastqc).

Trimmomatic version 0.39 was employed to trim reads after a quality drop below a mean of Q20 in a window of 5 nucleotides (1). Only reads of at least 15 nucleotides were cleared for subsequent analyses. Trimmed and filtered reads were aligned versus the Ensembl human genome version hg38 (ensembl release 101) using STAR 2.7.7a with the parameters "--outFilterMismatchNoverLmax 0.1 --alignIntronMax 200000" (2). The number of reads

aligning to genes was counted with featureCounts 1.6.5 from the Subread package (3). Only reads mapping at least partially inside exons were admitted and aggregated per gene. Reads overlapping multiple genes or aligning to multiple regions were excluded. Differentially expressed genes were identified using DESeq2 version 1.30.0 (4). Only genes with a minimum fold change of +- 1.5 (log2 +-0.59), a maximum Benjamini-Hochberg corrected p-value of 0.05, and a minimum combined mean of 5 reads were deemed to be significantly differentially expressed (DEG). The Ensemble annotation was enriched with UniProt data (release 24.03.2017) based on Ensembl gene identifiers (Activities at the Universal Protein Resource (UniProt)). DEGs were submitted to gene set enrichment analyses with KOBAS (5). The resulting plot shows pathways with P-Value < 0.05 (represented by dashed line). The raw data is provided in Gene Expression Omnibus under the accession number GSE130482.

Proteome Profiler Human Apoptosis Array

SW480 cells were transduced with either shSCRMBL as a control or with shTGM2-1 and shTGM2-2. Three days after transduction, cells were lysed on ice in M-PER Mammalian extraction Reagent and supplemented with Pierce Protease and Phosphatase Inhibitor Mini Tablets (Thermo Fisher Scientific). The Proteome Profiler Human Apoptosis Array kit (R&D Systems) procedure was performed as described in the manufacturer's instructions. This antibody-pair-based assay allows the detection of 43 human apoptotic markers. Briefly, protein sample was added to an antibody-spotted membrane, each pair of spots representing a different apoptotic marker, after overnight incubation at 4°C, membranes were washed followed by incubation with biotinylated detection antibodies and subsequently with streptavidin HRP. The protein spots were visualized using the ECL detection kit (GE Healthcare, Chicago, Illinois, USA) and the image was captured by Fusion FX7-Imaging system (Witec AG, Sursee, Switzerland). Quantification was performed using ImageJ Software.

Proximity ligation assay

Proximity ligation assay was performed using the Duolink in situ Starter kit (mouse/rabbit) from Merck (Darmstadt, Germany). Briefly, slides and filters were placed into the Double CytoSep (Simport, Quebec, Canada) according to the manufacturer's instructions. 50 000 SW480 cells or 50 000 patient-derived colon cancer and corresponding normal mucosa cells were used for every sample chamber. Cells were centrifuged at 500 g for 5 minutes. After centrifugation cells were fixed with 4% paraformaldehyde for 20 minutes at 4°C. After fixation the slides were washed with PBS, followed by blocking and permeabilization of cells using Duolink blocking solution (Merck) for 1.5 hours at 4°C in the humidity chamber. Slides were washed with PBS/0.2% Saponin at room temperature and primary antibodies were added. Primary antibodies (mouse anti-TGM2 antibody (CUB7402, Abcam) and rabbit anti-phospho p53(S15) antibody, (D4S1H, Cell Signaling)) were diluted 1:100 in Duolink antibody dilution reagent (Merck). On every spot 15 µl of primary antibody mix was added and incubated at 4°C overnight in a humidity chamber. The slides were washed in a staining jar for 5 minutes at room temperature in the following order; PBS/0.5%Tween20, PBS/0.2%Saponine/0.5%, Tween20, PBS/0.5%Tween20. The Duolink PLA probes (Merck) were diluted 1:5 according to the manufacturer's instructions and incubated for 60 minutes at 37°C in a humidity chamber. Before ligation, slides were washed as described before at room temperature. Ligation was performed according to the manufacturer's instructions and 15 µl ligase mix was added at each spot. After ligation, slides were washed in the order described before. The amplification mix was prepared according to the manufacturer's instructions and 15 µl amplification solution was added on each spot for 100 minutes at 37°C in a humidity chamber. After final washing, spots were mounted with mounting medium containing DAPI. Finally, the slides were analyzed using a fluorescence microscope.

Co-Immunoprecipitation

For Immunoprecipitations (IP), SW480, HCT-116 and HCT-116$^{p53-/-}$ cells were lysed in Pierce™ IP lysis buffer containing protease inhibitor, homogenized using Precellys® Evolution homogenizer. The protein extracts were centrifuged at 13 000 ×*g* for 20 minutes at 4°C. IP was performed using Dynabeads™ Protein G Immunoprecipitation kit (Thermo Fisher Scientific) according to manufacturer's instructions. Briefly, the antibody magnetic bead complex was prepared by adding 5µg of mouse anti-TGM2 antibody (CUB7402, Abcam) to the magnetic beads for 10 minutes at room temperature. Beads were washed twice with binding and washing buffer. Antigen was immunoprecipitated by adding the protein extracts to the magnetic antibody complex for an hour at 4°C in a rotator mixer. The beads (Magnetic ab-ag complex) were then washed 3x with washing buffer, followed by elution and denaturation of the target antigen at 70°C for 10 minutes. The eluted target protein was subjected to Simple Western technology (Bio-Techne, Wiesbaden, Germany) for protein detection of TGM2, p53, p53(S-15), p53(S46) and p53(S392) as described above.

Super-resolution microscopy

Super-resolution microscopy was conducted applying the concept of DNA points accumulation for imaging in nanoscale topography (DNA-PAINT) (6). Experiments were conducted in 8-well chambered coverslips (Sarstedt, Numbrecht, Germany) coated with fibronectin (Sigma-Aldrich) shortly before cell seeding to facilitate cell growth. 15 µg/mL fibronectin was incubated at 37°C for 1 hour and air-dried for an additional hour under sterile conditions. 100 000 SW480 and 80 000 HCT-116 cells per well were seeded and incubated overnight at 37°C. Cells were rinsed with PBS and subsequently fixed for 15 minutes using 4% methanol-free formaldehyde (Sigma-Aldrich). Cells were rinsed three times with PBS and incubated with the blocking buffer containing 2% bovine serum albumin and 0,1% Triton-X 100 in PBS for 1 hour at room temperature. Samples were subsequently incubated with the primary antibodies

(mouse anti-TGM2, 1:100, CUB7402, Abcam; rabbit anti-phospho p53(S15), 1:100, #AF1043 R&D Systems) in blocking buffer while shaking slowly at room temperature for two hours. Samples were rinsed three times with PBS. Oligonucleotide-labeled secondary antibodies (P1-goat anti-mouse IgG, P5-donkey anti-rabbit IgG) were prepared according to the protocol by Schnitzbauer et al. using a maleimide-PEG2-succinimidyl ester as molecular linker (Sigma-Aldrich, USA) (6). Thiol-modified oligonucleotides were ordered from Metabion (Planegg, Germany) and secondary antibodies were from Jackson ImmunoResearch (Ely, UK). The samples were incubated with DNA-labeled secondary antibodies diluted 1:100 in blocking buffer for 1 hour at room temperature while slightly shaking. The samples were rinsed three times for 5 minutes with PBS followed by post-fixation with 4% methanol-free formaldehyde (Sigma-Aldrich) in PBS for 10 minutes at room temperature. After three additional washing steps, fiducial markers were added to the samples to allow for drift correction and alignment of the TGM2 and p53 imaging channels. For this purpose, a solution of 100 nm gold nanoparticles (Nanopartz, USA) was sonicated for 10 minutes, diluted 1:10 in PBS and sonicated again for 10 minutes. 200 μL of the diluted nanoparticles were added to each chamber, incubated for 15 minutes at room temperature and subsequently rinsed three times with PBS. DNA imager strands P1 (5′ TAGATGTAT 3′) and P5 (5′ CATACATTGA 3′) (7), both labeled at the 3′-end with ATTO 655 (Eurofins Genomics, Ebersberg, Germany), were diluted in imaging buffer consisting of 500 mM NaCl, 1 mM EDTA and 3% (v/v) ethylene carbonate (8) (all Sigma-Aldrich) in 1x PBS (prepared from 10x stock, #D1408, Sigma-Aldrich) to final concentrations of 2 nM P1 and 3 nM P5 for measurements in HCT-116 cells as well as 4 nM P1 and 5 nM P5 for experiments in SW480 cells.

Exchange-PAINT experiments were performed on a commercial N-STORM microscope system (Nikon, Japan) following a previously published procedure (9, 10). The organic fluorophores were excited in total internal reflection fluorescence (TIRF) mode using a 647 nm laser operated at an irradiation density of 1.8 kW/cm^2. Data were acquired using an EMCCD

camera (DU-897U-CS0-#BV, Andor Technology, Ireland) with the following settings: 256 × 256 px image size, 157 nm pixel size, 150 EM gain, 1x preamp gain, 50 000 frames per movie, 20 ms frame time, 17 000 MHz readout rate and active frame transfer. LCControl (Agilent, USA), NIS elements (Nikon, Japan) and µManager (6) were used for setup control and data acquisition. After TGM2 imaging using P1-ATTO 655, samples were rinsed 8-10 times with imaging buffer to fully eliminate remaining imager strands followed by addition of the second imager strand P5-ATTO 655 to measure p53(S15) in the same cells. At least four cells were imaged and analyzed per condition.

Data were processed using Picasso (v. 0.2.8) (6). Single-molecule localizations from DNA-PAINT movies were identified and fitted in Picasso Localize with the integrated Gaussian maximum likelihood estimation algorithm using a baseline of 196, a sensitivity of 14.3, a camera quantum efficiency of 0.95 and a min net gradient in the range of 6500-8000 as localization parameters. Localization lists were imported into Picasso Render to reconstruct super-resolved images, and perform drift correction and alignment of the TGM2 and p53(S15) channels. Eventually, localizations were filtered for the localization precision ($lp_{x,y}$) as well as the width of the point spread functions ($s_{x,y}$) in x- and y-direction to remove signals originating from out-of-focus planes, unspecific binding or gold nanoparticles. Merged images of both proteins were generated from filtered localization lists.

References

1. Bolger AM, Lohse M, Usadel B. Trimmomatic: A flexible trimmer for Illumina sequence data. Bioinformatics 2014; 30(15):2114–20.

2. Dobin A, Davis CA, Schlesinger F, Drenkow J, Zaleski C, Jha S et al. STAR: Ultrafast universal RNA-seq aligner. Bioinformatics 2013; 29(1):15–21.

3. Liao Y, Smyth GK, Shi W. featureCounts: An efficient general purpose program for assigning sequence reads to genomic features. Bioinformatics 2014; 30(7):923–30.

4. Love MI, Huber W, Anders S. Moderated estimation of fold change and dispersion for RNA-seq data with DESeq2. Genome Biol 2014; 15(12):550.

5. Xie C, Mao X, Huang J, Ding Y, Wu J, Dong S et al. KOBAS 2.0: A web server for annotation and identification of enriched pathways and diseases. Nucleic Acids Res 2011; 39(Web Server issue):W316-22.

6. Schnitzbauer J, Strauss MT, Schlichthaerle T, Schueder F, Jungmann R. Super-resolution microscopy with DNA-PAINT. Nat Protoc 2017; 12(6):1198–228.

7. Jungmann R, Avendaño MS, Dai M, Woehrstein JB, Agasti SS, Feiger Z et al. Quantitative super-resolution imaging with qPAINT. Nat Methods 2016; 13(5):439–42.

8. Civitci F, Shangguan J, Zheng T, Tao K, Rames M, Kenison J et al. Fast and multiplexed superresolution imaging with DNA-PAINT-ERS. Nat Commun 2020; 11(1):4339.

9. Harwardt M-LIE, Schröder MS, Li Y, Malkusch S, Freund P, Gupta S et al. Single-Molecule Super-Resolution Microscopy Reveals Heteromeric Complexes of MET and EGFR upon Ligand Activation. Int J Mol Sci 2020; 21:2803.

10. Schröder MS, Harwardt M-LIE, Rahm JV, Li Y, Freund P, Dietz MS et al. Imaging the fibroblast growth factor receptor network on the plasma membrane with DNA-assisted single-molecule super-resolution microscopy. Methods 2020; S1046-2023(20)30024-4.
